# DTL-DephosSite: Deep Transfer Learning Based Approach to Predict Dephosphorylation Sites

**DOI:** 10.3389/fcell.2021.662983

**Published:** 2021-06-24

**Authors:** Meenal Chaudhari, Niraj Thapa, Hamid Ismail, Sandhya Chopade, Doina Caragea, Maja Köhn, Robert H. Newman, Dukka B. KC

**Affiliations:** ^1^Department of Computational Data Science and Engineering, North Carolina A&T State University, Greensboro, NC, United States; ^2^Department of Computer Science, Kansas State University, Manhattan, KS, United States; ^3^Faculty of Biology, Signalling Research Centres BIOSS and CIBSS, University of Freiburg, Freiburg, Germany; ^4^Department of Biology, North Carolina A&T State University, Greensboro, NC, United States; ^5^Department of Electrical Engineering and Computer Science, Wichita State University, Wichita, KS, United States

**Keywords:** post-translational modification, deep learning, transfer learning, dephosphorylation, computational prediction

## Abstract

Phosphorylation, which is mediated by protein kinases and opposed by protein phosphatases, is an important post-translational modification that regulates many cellular processes, including cellular metabolism, cell migration, and cell division. Due to its essential role in cellular physiology, a great deal of attention has been devoted to identifying sites of phosphorylation on cellular proteins and understanding how modification of these sites affects their cellular functions. This has led to the development of several computational methods designed to predict sites of phosphorylation based on a protein’s primary amino acid sequence. In contrast, much less attention has been paid to dephosphorylation and its role in regulating the phosphorylation status of proteins inside cells. Indeed, to date, dephosphorylation site prediction tools have been restricted to a few tyrosine phosphatases. To fill this knowledge gap, we have employed a transfer learning strategy to develop a deep learning-based model to predict sites that are likely to be dephosphorylated. Based on independent test results, our model, which we termed DTL-DephosSite, achieved efficiency scores for phosphoserine/phosphothreonine residues of 84%, 84% and 0.68 with respect to sensitivity (SN), specificity (SP) and Matthew’s correlation coefficient (MCC). Similarly, DTL-DephosSite exhibited efficiency scores of 75%, 88% and 0.64 for phosphotyrosine residues with respect to SN, SP, and MCC.

## Introduction

**FIGURE 1:**
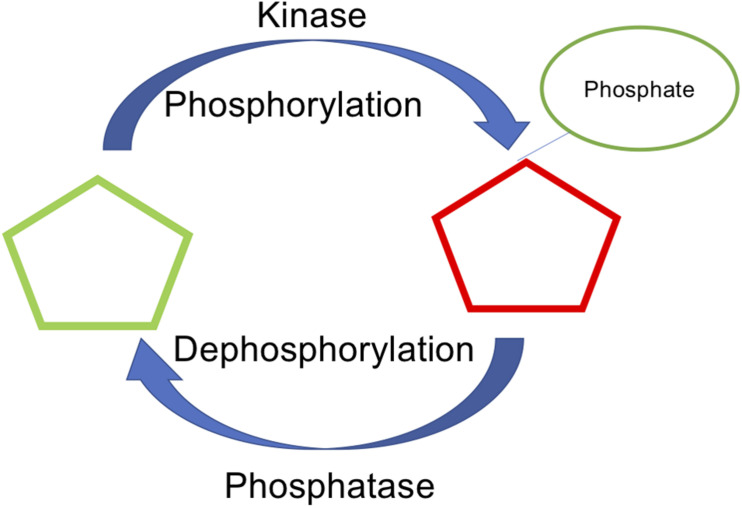
Phosphorylation and dephosphorylation, mediated by kinase and phosphatase as a key reversible post translational modification.

Protein phosphorylation is an important posttranslational modification (PTM) that regulates many cellular activities and contributes to the etiology and progression of several pervasive diseases, including cancer, diabetes, cardiovascular disease, and neurodegeneration. In eukaryotic cells, phosphorylation, and subsequent dephosphorylation, occurs on serine (S), threonine (T), and tyrosine (Y) residues located on the protein surface. To date, more than two-thirds of the ∼21,000 proteins encoded by the human genome have been shown to be phosphorylated, making phosphorylation one of the most wide-spread and broadly studied protein PTMs (Ardito et al., 2017). The precise regulation of the phosphorylation status of a protein depends on the opposing activities of protein kinases, which catalyze the transfer of the γ-phosphate of ATP to their downstream substrates, and protein phosphatases, which catalyze the dephosphorylation (i.e., removal of the phosphate group) from the modified site (Figure 1). While it is often assumed that any site that can be phosphorylated can also be dephosphorylated, this may not always be the case (Bechtel et al., 1977; Bornancin and Parker, 1997; Keshwani et al., 2012; Senga et al., 2015). Similarly, certain sites may be dephosphorylated more efficiently than others. Though rare, there are instances of phosphorylation sites that are resistant to dephosphorylation. For instance, once phosphorylated, both T197 and S338 in cAMP-dependent protein kinase (PKA) are resistant to dephosphorylation (Bechtel et al., 1977; Keshwani et al., 2012). Similarly, protein kinase G (PKG), protein kinase C (PKC), and calcium/calmodulin-dependent protein kinase 1δ (CAMK1δ) each exhibit phosphatase-resistant states (Bornancin and Parker, 1997; Keshwani et al., 2012; Senga et al., 2015). The relative efficiency of dephosphorylation at a particular site may be, at least partially, dependent on the local protein environment and the ability of phosphatases to recognize the phosphosite.

Phosphorylation site prediction has recently emerged as an important problem in the field of bioinformatics. As a result, many phosphorylation site prediction tools have been developed to predict both general and kinase-specific phosphorylation sites (Lumbanraja et al., 2019; Luo et al., 2019; Haixia et al., 2020; Wang D. et al., 2020; Ahmed et al., 2021; Guo et al., 2021). For instance, to predict general phosphorylation sites based on the primary amino acid sequence of an input protein, Ismail et al. developed the Random Forest (RF)-based phosphosite predictor 2.0 (RF-Phos 2.0) (Ismail et al., 2016). RF-Phos 2.0 assesses the relative importance of hand-selected features to identify putative sites of phosphorylation across many protein families. More recently, Luo et al. developed Deep-Phos, a general and kinase-specific phosphorylation site predictor based on multilayer convolutional neural networks (CNN) (Luo et al., 2019).

While many phosphorylation site prediction tools have been developed over the past decade to identify putative sites of S, T, and Y phosphorylation (Ismail et al., 2016; Luo et al., 2019; Wang D. et al., 2020), computational prediction of dephosphorylation sites has been much more limited (Wang et al., 2016). Information about dephosphorylation sites is important because it can provide insights into the molecular determinants of phosphatase recognition and may offer clues about the biological half-life of a given phosphorylation event. To date, computational methods for dephosphorylation site prediction have focused on a relatively small group of tyrosine phosphatases consisting of protein tyrosine phosphatase 1B (PTP1B) and the Src homology 2 (SH2) domain-containing phosphatases, SHP-1 and SHP-2 (Wu et al., 2014; Wang et al., 2016; Jia et al., 2017). For instance, Wu et al. developed a method that uses the k-nearest neighbor algorithm to identity the substrate sites of PTP1B, SHP-1, and SHP-2 based on the sequence features of manually collected dephosphorylation sites (Wu et al., 2014). Meanwhile, Wang et al. developed two sophisticated models for predicting the substrate dephosphorylation sites of these phosphatases. The first model, which they termed MGPS-DEPHOS, is modified from the Group-based Prediction System (GPS) while the second model, termed CKSAAP-DEPHOS, utilizes a combination of support vector machine (SVM) and the k-spaced amino acid pairs (CKSAAP) encoding scheme. Finally, Jia et al. (2017) combined the sequence-based bi-profile Bayes feature extraction technique and SVM to predict sites for the same three phosphatases.

One of the primary reasons for the proliferation of phosphorylation site predictors over the past decade is the availability of large databases cataloging experimentally identified phosphorylation sites, such as PhosphoSitePlus and PhosphoELM (Dinkel et al., 2011; Hornbeck et al., 2019). Unfortunately, similar databases have not been available for dephosphorylation sites. However, with the recent curation of the DEPOD database of S, T, and Y dephosphorylation sites, the development of dephosphorylation site predictors is now feasible (Damle and Köhn, 2019). In this study, we compiled a dataset of S, T, and Y dephosphorylation sites from the DEPOD database (Damle and Köhn, 2019) and further extended the available dataset through literature mining, increasing the database more than threefold. We then developed a transfer learning approach utilizing the phosphorylation dataset and a bidirectional long short-term memory (Bi-LSTM) deep learning-based model to predict dephosphorylation sites on proteins. To our knowledge, this is the first study to develop a general dephosphorylation predictor for Y residues and the first to predict general dephosphorylation sites for S/T residues. Our models, which we termed DTL-DephosSite-ST and DTL-DephosSite-Y, performed well when assessed using both five-fold cross-validation and an independent test set.

Here we have developed the first general phosphatase site prediction tool. Unlike phosphatase-specific methods, which are designed to predict both the site of dephosphorylation and the phosphatase mediating the dephosphorylation event, our general dephosphorylation site prediction method is able to identify putative sites of dephosphorylation irrespective of the phosphatase mediating the dephosphorylation event. This is analogous to the results obtained by MS/MS-based experiments, where information about the responsible phosphatase is not known. Importantly, phosphatase-specific methods are currently restricted to predictions for only three phosphatases (i.e., PTP1B, SHP1, and SHP2), which represent a very small fraction of phosphatases encoded by the human genome. This is likely due, in part, to limited information about the specific phosphatase that mediates a given dephosphorylation event. Therefore, general dephosphorylation site prediction methods offer distinct advantages when the primary goal is to predict whether or not a given site is dephosphorylated.

## Materials and Methods

### Datasets

The human DEPhOsphorylation Database, DEPOD, is a database of dephosphorylation sites that was recently expanded in an updated version in 2019 (Damle and Köhn, 2019). DEPOD accounts for 241 active and 13 inactive human phosphatases in total. Among the active phosphatases, 194 include substrate data. This database provided the starting point to create dephosphorylation datasets for S, T, and Y residues. To this end, we collected all the FASTA sequences from the UniProt database (UniProt Consortium, 2019) and extracted windows with the targeted S/T/Y residue at the center and 16 residues on each side. Negative sequences were extracted using all S/T/Y residues except those that are known positive sites (i.e., all residues except those sites that are known to be dephosphorylated). During the generation of sequences, no fillers (i.e., “-”) were used. To minimize the loss of sequences occurring at the ends, a maximum window size of 33 was chosen. Any redundant sequences within and between the positive and negative sites were removed to obtain a non-redundant set. Similar to our previous studies (Chaudhari et al., 2020; Thapa et al., 2020), we used an under-sampling strategy to balance the dataset, which had more negative sites than positive sites prior to balancing (Aridas GLitaFNaCK, 2017). Under-sampling allows random selection of negative sequences to make the number of negative sites equal to the number of positive sequences, thus balancing the dataset.

Once constructed, the dataset was further divided into training and test sets, such that 80% of the data was used to train the models and the remaining 20% of the data was kept aside for independent testing. This training-test dataset, which we termed the DEPOD-19 dataset (Table 1), consists of 133 positive sites for S, 58 positive sites for T, and 101 positive sites for Y (Table 1).

**TABLE 1:** Summary of the training and test datasets used for model development based on sites extracted from the DEPOD-19, Downreg (literature resources) and composite ComDephos datasets.

Dataset	Residue	Train	Test	Total positive	Total negative
DEPOD-19	ST	304	78	191	191
	Y	161	41	101	101
Downreg	ST	1478	370	924	924
	Y	40	10	25	25
ComDephos	ST	1,806	446	1,112	1,112
	Y	201	50	125	125

Though phosphorylation is one of the most wide-spread and well-studied PTMs in eukaryotes, comprehensive lists of dephosphorylation sites are scarce. This is likely due to the lack of computational studies in the field and technical challenges associated with the detection of dephosphorylation sites. Therefore, in order to enlarge the dephosphorylation site dataset (Damle and Köhn, 2019), we did a comprehensive literature review to identify phosphorylated sites that were down-regulated in cells following treatment with various agents. For a given site to be considered dephosphorylated, there must have been no co-stimulation during treatment and the analysis must have been conducted less than an hour after stimulation (to prevent changes in protein expression from substantially contributing to the observed changes in phosphorylation state). Moreover, because many phosphorylation sites have been identified in human cells, we only considered publications using human cells. Finally, to avoid errors stemming from heterogeneity in the phosphorylation patterns in different phases of the cell cycle, our analyses only included cells that had been arrested in the mitotic phase. Using these criteria, we developed the “Downreg” dataset, which consists of 949 dephosphorylation sites in 624 proteins. These included 772 S, 152 T, and 25 Y residues, which represents an ∼3.25-fold increase relative to the DEPOD-19 dataset, as summarized in Table 1 and Supplementary Table 2. A summary of the data sources and the corresponding descriptive statistics for each study (e.g., false discovery rate and data distribution) are included in Supplementary Table 1 and all the newly added dephosphorylation sites from the “Downreg” dataset have been added in Supplementary Table 12.

During sequence extraction, a sub-sequence with window size of 33 centered around the site of interest was created in a manner similar to that described for the DEPOD-19 dataset above. Supplementary Table 1 summarizes the literature sources and the number of dephosphorylation sites identified. Removal of common sequences within and between the positive and negative sets was performed to obtain a non-redundant dataset. Finally, the “combined dephosphorylation site” dataset was obtained by merging the DEPOD-19 and Downreg datasets and removing any duplicate protein sequences of window size 33. The combined dephosphorylation dataset (ComDephos) is summarized in Table 1. For model development, the DEPOD-19 and the ComDephos datasets were used.

### Bidirectional LSTM Model

Long Short-Term Memory (LSTM) models are known to provide good performance with sequence data (Hochreiter and Schmidhuber, 1997). LSTM uses different memory cells and an additive gradient function helps to overcome the vanishing and exploding gradient problems in recurrent neural networks (RNN). Importantly, the use of memory cells can keep sequence information in the network for long periods of time.

**FIGURE 2:**
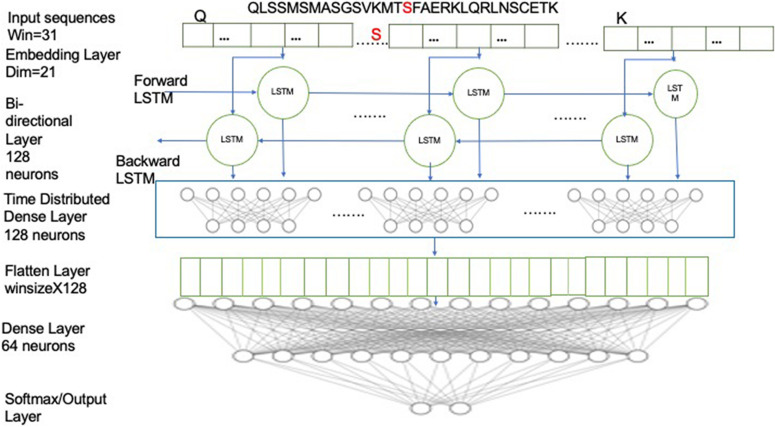
Schematic illustrating the Bi-LSTM deep learning architecture and the parameters used. The input sequence is first fed into embedding layer with dimension of 21, then through two Bi-LSTM layers with 128 neurons and then followed by a time-distributed layer of 128 neurons, which was followed by a flatten layer and then followed by dense layer with 2 neurons with softmax activation.

A single LSTM cell consists of three gates: “input,” “forget,” and “output” gates (Figure 2). The input layer (z_*t*_) consists of a sigmoid layer and a tanh layer. The sigmoid layer filters the previous state to select the relevant cell states for the context while the tanh layer provides a range of values to take to the selected states. The forget layer (r_*t*_) consists of a sigmoid layer, which filters the irrelevant previous cell states by dropping them out. The output layer (hþ_*t*_) employs a tanh layer to provide an update to the selected states, as provided by the input layer (Hochreiter and Schmidhuber, 1997).

The forget gate layer takes previous hidden cells and inputs for each previous cell state. The sigmoid node in the forget gate adds in 0 or 1 to the previous hidden state, deciding whether it would be passed over to the next hidden state. The input gate layer has sigmoid and tanh nodes, where the sigmoid acts as a selection node and selects the values that need to be updated. Meanwhile, the tanh nodes provide a vector of new candidate values for the selected states, acting as the update node. Finally, the output is obtained by adding previous values for old states and updated values for the selective nodes.

In this architecture, we have employed a bidirectional LSTM layer (Bi-LSTM), which uses twice the number of neurons as a conventional LSTM layer. The double neurons create two sets of networks, moving in both the forward and the reverse directions (Schuster and Paliwal, 1997). Thus, a Bi-LSTM layer is able to predict the context of the target residue from the residues from both directions. For example, given a window sequence:

NYTPTSPNYSPTSPSY
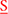
PTSPSYSPTSPSYSPS

where the S (red) in the center represents the target residue, the forward LSTM network would predict the probability of having S, given the knowledge of the residues preceding it (i.e., “NYTPTSPNYSPTSPSY”) while the backward/reverse LSTM network would predict the probability of having S, given the knowledge of residues following it (i.e., “PTSPSYSPTSPSYSPS”). The window sequences were integer encoded, such that each character in the sequence was replaced by its corresponding integer value. The integer encoded sequences were then fed to the embedding layer, which provides an embedding dimension of 21, which is known to be optimal based on our previous studies (Chaudhari et al., 2020; Thapa et al., 2020). The embedding layer helps in capturing the latent representation of the encodings using a look-up table (Keras, 2015). For model development, a Bi-LSTM layer with 128 neurons was used, with timesteps equivalent to the window size, and return sequences kept as “true.” Next, it was followed with a time-distributed layer of 128 neurons. The time-distributed layer applies dense layer operation to every timestep of the 3D tensor (Keras, 2015). This was followed by a flatten layer with a dropout of 0.4 to avoid overfitting and a dense layer of 64 neurons, which was then followed by the output dense layer with 2 neurons with softmax activation. The model was compiled on binary cross-entropy loss using the Adam optimizer (Kingma and Ba, 2014). We used two callbacks while fitting the model: ModelCheckpoint and reduce learning rate on Plateau. ModelCheckpoint obtains the best model with respect to validation accuracy while the reducing learning rate helps in learning the parameters better, especially when the data size is small (Li and Hoiem, 2018). Parameters have been optimized to the settings shown in Table 2.

**TABLE 2:** Parameters used in LSTM Model for dephosphorylation.

Parameters	Settings
Embedding output dimension	21
Learning rate	0.01
Batch size	512
Epochs	30
LSTM_layer1_neurons	128
Dropout	0.4
Dense_layer_neurons	128, 64, 2

### Transfer Learning

As molecular counterparts, phosphorylation and dephosphorylation are closely related to one another but the cellular enzymes catalyzing each event (as well as the molecular determinants underlying recognition of the sites) are different. Moreover, the extensive study of phosphorylation sites has resulted in a comparatively large dataset of phosphosites, while the amount of information about dephosphorylation events has led to a relatively sparse dataset. Taken together, these observations suggest that a transfer learning strategy could be applied to dephosphorylation site prediction.

Recently, deep learning has been used to solve various problems in bioinformatics (Li et al., 2019; Tang et al., 2019; Chaudhari et al., 2020; Thapa et al., 2020; Wang D. et al., 2020; Wang Y. et al., 2020). One of the most serious problems associated with deep learning stems from data dependence. For instance, a significant challenge is posed by the lack of labeled data for the task-of-interest, e.g., dephosphorylation. Indeed, the problem of insufficient training data is an inescapable problem in various areas of bioinformatics. For dephosphorylation, the expense of data acquisition makes it particularly difficult to construct a large-scale, well-annotated dataset.

Previous studies suggest that, when trained on images, deep learning networks tend to learn first-layer features that do not appear to be specific to a particular task (Yosinski et al., 2014). Such first layer features are general in that they are applicable to many datasets and tasks. Exploiting this fact, transfer learning relaxes the hypothesis that the training data and test data are not required to be “independently and identically distributed” and that the model in the target domain does not need to be trained from scratch, which can significantly reduce the burden of training data size (Tan et al., 2018). Transfer of knowledge through shared parameters and weights of the source model and the target domain is one of the strategies in transfer learning (Weiss et al., 2016).

With the exception of a handful of dual specificity kinases and phosphatases, most kinases and phosphatases recognize either S/T or Y residues. Therefore, as is common in phosphorylation site prediction, we considered two models: one for S and T residues and another for Y residues. Thus, distinct phosphorylation and dephosphorylation datasets were formed and designated the Phos-ST and Phos-Y datasets and the Dephos-ST and Dephos-Y datasets.

**FIGURE 3:**
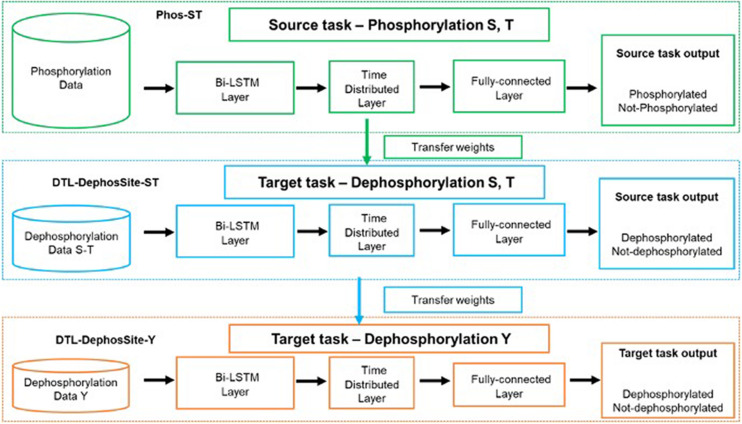
Schematic illustrating the transfer-learning. Green dotted box depicts the training on source task, phosphorylation (S,T), to obtain the Phos-ST model. Once the Phos-Model was obtained the Bi-LSTM model was instantiated with the Phos-Model weights before being trained on the dephosphorylation data. Blue dotted box depicts the transfer learning on the target task, dephosphorylation for ST residues, to obtain the DTL-DephosSite-ST model. During transfer learning, all layers were allowed to re-train and none of the layers were frozen. (We tried various options with various layers frozen but this version produced the best results). Orange dotted box depicts the transfer learning from DTL-DephosSite-ST, to obtain the DTL-DephosSite-Y model.

During transfer learning, three important questions need to be answered: (a) what to transfer, (b) when to transfer, and (c) how to transfer. Therefore, to allow our framework to accommodate smaller datasets, we applied a two-step transfer learning scheme that included a pre-training step and a fine-tuning step (Figure 3). The pre-training step results in a source model, which is then available to adapt on the target dataset through fine-tuning.

The pre-training step involves the training of our Bi-LSTM model (as described in section “Bidirectional LSTM Model”) on the available phosphorylation data (Wang D. et al., 2020), which are provided in Supplementary Table 3. This resulted in a Phos-model that contains learned weights to classify a given motif as phosphorylated or not, specifically the S/T residues. During the fine-tuning step, the weights learned by the source Phos-model were transferred to a new instance of the Bi-LSTM architecture. The model was then trained on the Dephos data containing the S/T residues in the center, thus obtaining a transfer-learned Dephos model for S/T residues. We experimented with different combinations of frozen and re-trained layers and identified a model, where all layers are allowed to re-train, that learned better than others.

Similarly, for the prediction of Y dephosphorylation sites, we experimented with performing transfer learning from Phos-ST-to-Dephos-Y as well as Phos-Y-to-Dephos-Y. These studies suggested that the Dephos-ST-to-Dephos-Y transfer worked the best. Thus, the pre-training step involved training the Dephos-ST model, initialized with transfer-learned weights from Phos-ST on the Dephos-ST dataset. During the fine-tuning step, we retrained all layers on the Dephos-Y dataset. Though varying the layers that were kept frozen or re-trained had less impact in performance, retraining all layers helped in attaining more consistent results.

Finally, we also employed the transfer learned Dephos-Y model on the available phosphatase specific datasets (Wang et al., 2016) for PTP1B, SHP1, and SHP2 (Supplementary Table 9).

### Performance and Evaluation

To evaluate the performance of each model, we used a confusion matrix to determine Sensitivity (SN), Specificity (SP), Accuracy (ACC) and the Receiver Operating Characteristic (ROC) curve as the performance metrics. The models were evaluated using five-fold cross-validation on the benchmark training dataset and an independent test set.

ACC describes the correctly predicted residues out of the total residues (Eq. 1). Meanwhile, SN defines the model’s ability to distinguish positive residues (Eq. 2) and SP measures the model’s ability to correctly identify the negative residues (Eq. 3). Matthews Correlation Coefficient (MCC) is the calculated score that takes into account the model’s predictive capability with respect to both positive and negative residues (Eq. 4). Likewise, the ROC curve provides a graphical representation of the diagnostic ability of the classifier. The area under the ROC curve (AUC) is used to compare various models, with the models having the highest AUC scores generally performing better in classification than those with lower AUC scores.

(1) $$A\,c\,c\,u\,r\,a\,c\,y=\frac{T\,P+T\,N}{T\,P+T\,N+F\,P+F\,N}\times 100$$

(2) $$S\,e\,n\,s\,i\,t\,i\,v\,i\,t\,y=\frac{T\,P}{T\,P+F\,N}\times 100$$

(3) $$S\,p\,e\,c\,i\,f\,i\,c\,i\,t\,y=\frac{T\,N}{T\,N+F\,P}\times 100$$

(4) $$M\,C\,C=\frac{\left(T\,P\right)\,\left(T\,N\right)-\left(F\,P\right)\,\left(F\,N\right)}{\sqrt{\left(T\,P+F\,P\right)\,\left(T\,P+F\,N\right)\,\left(T\,N+F\,P\right)\,\left(T\,N+F\,N\right)}}$$

## Results and Discussion

### Bidirectional Model on Dephos Datasets (Without Transfer Learning)

To efficiently identify sites that are likely to be dephosphorylated in proteins, we sought to develop a dephosphorylation site prediction tool using the recently expanded DEPOD-19 dataset (Table 1). To this end, we first extracted FASTA sequences from the DEPOD-19 dataset. During extraction, we limited the window size to 33 in order to minimize the loss of sequences at the ends of the sequences. We then applied a bidirectional long short-term memory (Bi-LSTM) deep learning strategy to the dataset. During these analyses, we trained on the train dataset and the performance of the resulting model was evaluated using an independent test set (representing 20% of the original dataset) that was kept aside from the training set. These analyses suggest that our preliminary model had reasonable sensitivity (SN) and receiver operating characteristic (ROC) scores of 0.85 and 0.79, respectively. However, this preliminary model suffered with respect to specificity (SP) and Matthew’s correlation coefficient (MCC), where it exhibited scores of 0.49 and 0.36, respectively (Table 3). A feature-based machine learning strategy employing random forest (RF) yielded similar results (Supplementary Table 4).

**TABLE 3:** Performance of Deep learning model on Depod19 and ComDephos datasets.

Dataset	MCC	Specificity	Sensitivity	ROC_AUC
Depod19	0.36	0.49	0.85	0.79
ComDephos	0.46	0.71	0.76	0.81

**Independent test results using the DEPOD-19 and the ComDephos datasets for ST residue.**

Though the DEPOD-19 dataset has recently been expanded to include 584 total sites, it still represents a relatively small dataset for model development using machine learning strategies. Therefore, to further expand the dataset, we conducted a comprehensive literature search for dephosphorylation sites. This yielded an additional 1,898 sites whose phosphorylation status decreased within an hour of treatment in mitotically arrested cells (Table 1; see section “Materials and Methods” for details). Combining this so called “Downreg” dataset with those sites that had already been curated in the DEPOD-19 dataset resulted in a composite “ComDephos” dataset containing 2,503 total, non-redundant dephosphorylation sites (composed of 1,806 S, 446 T, and 251 Y sites) (Table 1). We then repeated our Bi-LSTM-based learning scheme using the newly developed ComDephos dataset and assessed performance based on our independent test set (Table 3 and Supplementary Table 5). This led to marginal improvements in model performance using the independent datasets. For instance, while ROC increased marginally (2.5%), SP increased by 44.8% and SN decreased by 10.5%. Together, these changes resulted in a 27.8% increase in overall model performance, as assessed by MCC.

The observed gains are likely due to an increase in the size of the dataset, consistent with several reports that suggest that deep learning models perform well on large datasets and that an increase in the size of the dataset can increase the performance of the resulting model (Zhao, 2017; Feng et al., 2019). However, despite these gains, performance of the model developed using the ComDephos dataset was still relatively poor. Therefore, we asked if model performance could be enhanced using a transfer learning strategy.

### Development of S/T Dephosphorylation Site Predictor Using Transfer Learning on the Phosphorylation Site Database

In contrast to dephosphorylation sites, phosphorylation sites have been extensively annotated, totaling 484,110 sites in 20,217 proteins (PhosphoSitePlus; Hornbeck et al., 2019), as 1/31/2021). Given the inherent similarities in the physiochemical properties of the modified sites and the potential differences in the molecular determinants used by kinases and phosphatases to recognize sites of phosphorylation and dephosphorylation, respectively, we reasoned that a transfer learning approach could be applied to develop a model to predict sites of dephosphorylation (Figure 3). Therefore, we used the phosphorylation dataset described by Wang et al. (2017). This dataset, which is composed of 31,944 experimentally determined phosphorylation sites and an equal number of negative sites (i.e., S, T, or Y residues that are not known to be phosphorylated), was used to generate a source model (Supplementary Table 3). First, we explored the effect that window size had on phosphosite prediction. To this end, progressively smaller window sizes were created, starting with a window size of 33. This was achieved by removing one residue from each end of the sequence in successive steps to yield windows of 33, 31, 29, 27, 25, and 23. We then trained the Bi-LSTM model on the phosphorylation training dataset using each window size and tested on the independent test set (Supplementary Table 6). This led to our source phosphorylation model (Phos-Model) for their respective windows, which was used for transfer learning to the target dephosphorylation dataset.

Next, to apply the knowledge gained from phosphorylation site prediction to dephosphorylation, the Bi-LSTM model was instantiated with the Phos-Model weights before being trained on the DEPOD-19 and ComDephos datasets. During transfer learning, all layers were allowed to re-train in the fine-tuning step. This yielded a transfer-learned dephosphorylation model for each window size. To determine the optimal window size, we then conducted five-fold cross-validation of the transfer-learned dephosphorylation dataset based on the ComDephos dataset (Table 4). These analyses suggested that window sizes of 29 and 31 led to the best predictors based on MCC. A similar trend was also observed for the phosphorylation dataset (Supplementary Table 6) and for a transfer-learned model trained on the DEPOD-19 dataset (Supplementary Table 7). Since a window size of 31 performed marginally better with respect to SN and ROC, we selected this window for further analysis. We termed this transfer learned, deep learning-based S/T dephosphorylation site predictor, DTL-DephosSite-ST. Importantly, compared to the S/T model developed using deep learning alone, DTL-DephosSite-ST exhibited an increase in all performance metrics. This resulted in an ∼3.26-fold increase in overall performance for S/T, as assessed by MCC. Likewise, using our independent dataset, DTL-DephosSite-ST outperformed similar transfer-learned dephosphorylation site prediction models that had been trained using either different deep learning architectures, such as conventional LSTM or CNN, or the recently developed DeepPhos (Luo et al., 2019) phosphorylation site predictor, which utilizes densely connected CNNs (Table 5). Taken together, these data suggest that DTL-DephosSite-ST effectively predicts putative sites of dephosphorylation on S/T residues.

**TABLE 4:** Five-fold cross-validation of various window sizes for prediction of S/T residues following transfer learning using Phos-Model (source) and ComDephos dataset (target).

Window size	MCC ± SD	Specificity ± SD	Sensitivity ± SD	Accuracy ± SD	ROC_AUC
23	0.58 ± 0.05	0.78 ± 0.04	0.80 ± 0.01	0.79 ± 0.02	0.86
25	0.60 ± 0.04	0.78 ± 0.02	0.82 ± 0.03	0.80 ± 0.02	0.86
27	0.60 ± 0.05	0.79 ± 0.04	0.81 ± 0.02	0.80 ± 0.02	0.87
29	**0.61 ± 0.04**	**0.79 ± 0.02**	0.82 ± 0.03	0.80 ± 0.02	0.86
31	**0.61 ± 0.04**	0.77 ± 0.03	**0.83 ± 0.03**	0.80 ± 0.02	**0.87**
33	0.60 ± 0.05	0.78 ± 0.04	0.82 ± 0.03	0.80 ± 0.02	0.87

*The highest scores in each metric are highlighted in boldface.*

**TABLE 5:** Comparison between DTL-DephosSite-ST and transfer-learned models developed using other deep learning architectures based on an independent test set.

Architecture	MCC	Specificity	Sensitivity	ROC_AUC
CNN	0.60	0.74	**0.86**	0.89
LSTM	0.64	0.79	0.85	0.86
DeepPhos (DC-CNN): (Luo et al., 2019)	0.64	0.82	0.83	0.89
DTL-DephosSite-ST (Bi-LSTM)	**0.68**	**0.84**	0.84	**0.90**

**CNN, Convolutional Neural Network; LSTM, Long short-term memory; DC-CNN, Densely connected CNN; Bi-LSTM, bidirectional LSTM. The highest scores in each metric are highlighted in boldface.**

### Transfer Learning Dephos-Y

With a transfer-learned S/T dephosphorylation site model in hand, we used a similar strategy to identify putative sites of Y dephosphorylation. Specifically, transfer learning was applied to the Y residues in the ComDephos dataset using DTL-DephosSite-ST as the source model. To obtain the DTL-DephosSite-Y, the model was instantiated with the weights of DTL-DephosSite-ST and all layers were re-trained on the ComDephos-Y dataset. Similar to the results for the S/T models, five-fold cross-validation suggested that window sizes of 27 and 31 performed the best, with a window size of 31 exhibiting slightly higher values for the majority of performance metrics (Table 6). Interestingly, models that were trained in the same manner using the smaller DEPOD-19 dataset resulted in a more sporadic distribution across windows, with a window size of 27 achieving the best specificity, and a window size of 31 producing the highest values for MCC and Sensitivity (Supplementary Table 7). Such a sporadic distribution may suggest that we are approaching a lower limit with respect to the size of the dataset, beyond which transfer learning becomes less effective.

**TABLE 6:** Five-fold cross-validation of various window sizes for prediction of Y residues following transfer learning using DTL-DephosSite-ST (source) and ComDephos dataset (target).

Window size	MCC ± SD	Specificity ± SD	Sensitivity ± SD	Accuracy ± SD	ROC_AUC
23	0.53 ± 0.09	0.76 ± 0.11	0.76 ± 0.07	0.76 ± 0.04	0.81
25	0.49 ± 0.13	0.76 ± 0.12	0.72 ± 0.09	0.74 ± 0.06	0.79
27	**0.59 ± 0.06**	0.78 ± 0.10	**0.80 ± 0.09**	0.79 ± 0.03	0.82
29	0.50 ± 0.07	0.74 ± 0.06	0.76 ± 0.06	0.75 ± 0.03	0.82
31	**0.59 ± 0.10**	**0.83 ± 0.09**	0.76 ± 0.06	**0.80 ± 0.05**	**0.83**
33	0.58 ± 0.08	0.78 ± 0.07	**0.80 ± 0.04**	0.79 ± 0.05	0.82

*The highest scores for each metric are highlighted in boldface.*

Similarly, models that were trained using different combinations of source models and target datasets (e.g., Phospho-Y as source and ComDephos as target or Phospho-Y as source and DEPOD-19 as target) yielded models that performed well in most metrics, but not as well as the window size 31 Y dephoshorylation model generated using DTL-DephosSite-ST as the source model and the ComDephos dataset as the target dataset (Supplementary Table 8). For instance, window sizes of 27 and 31 exhibited similar MCC, with window size of 31 achieving the best specificity, accuracy and ROC scores. Therefore, we chose this model, which we named DTL-DephosSite-Y, for further analysis. Similar to DTL-DephosSite-ST, the newly developed DTL-DephosSite-Y performed well when evaluated using an independent test set (Table 7).

**TABLE 7:** Independent test results of DeepPhos (Luo et al., 2019), DTL-DephosSite-ST and DTL-DephosSite-Y on ComDephos independent set, using the optimized parameters.

Predictor	MCC	Specificity	Sensitivity	Accuracy	ROC_AUC
DeepPhos	0.44	0.48	**0.92**	0.70	0.86
DTL-DephosSite-ST	**0.68**	0.84	0.84	**0.84**	**0.90**
DTL-DephosSite-Y	0.64	**0.88**	0.75	0.82	0.89

**Here, results of DeepPhos model is provided to show the performance of a model trained on just Phosphorylation sites. The highest scores in each metric are highlighted in boldface.**

## Conclusion

Here, we describe a strategy that combines deep learning with transfer learning to develop general dephosphorylation site predictors of S/T and Y residues. To our knowledge, the resulting models, termed DTL-DephosSite-ST and DTL-DephosSite-Y, are the first general dephosphorylation site predictors for S/T and Y dephosphorylation, respectively. Deep learning-based models have recently been developed for several important PTMs, including phosphorylation, methylation, acetylation, and succinylation, to name a few (Wang et al., 2017; Luo et al., 2019; Wu et al., 2019; Al-barakati et al., 2020; Chaudhari et al., 2020; Thapa et al., 2020; Ahmed et al., 2021). Similar to previous deep learning-based models, our models did not require any hand selected features during model development. However, unlike many of the other deep learning-based models that were developed using extensive PTM data, the number of experimentally identified dephosphorylation sites was relatively low. As a consequence, our initial attempts to develop dephosphorylation site predictors based solely on deep learning yielded models that did not predict sites efficiently. This is consistent with reports that deep learning does not perform as well on small datasets (Zhao, 2017; Feng et al., 2019). To overcome this limitation, we developed a transfer learning-based approach. Specifically, we generated a source model based on knowledge gained about phosphorylation using a Bi-LSTM deep learning architecture and then applied this information to the ComDephos dataset using transfer learning. The resulting models performed markedly better than those developed using Bi-LSTM alone. This suggests that our approach is able to learn solely through the patterns of motif sequences. Importantly, by utilizing a transfer learning-based strategy, we were able to capitalize on the richness of phosphorylation site datasets in order to improve the efficacy of dephosphorylation prediction. This provides an attractive solution to the scarce data problem and may be applicable in the development of other PTM predictors.

During this project, we also expanded the DEPOD-19 dephosphorylation dataset 3.25-fold to create computational datasets of dephosphorylation. Importantly, this study relies upon the correlation between the cellular processes of phosphorylation and dephosphorylation. We have attempted to measure the level of transferability between phosphorylation and dephosphorylation. Similar correlations are also likely to be found for other PTMs where the forward and reverse reactions are catalyzed by different classes of enzymes, such as methylation/demethylation and acetylation/deacetylation. Prediction of sites of these modifications may thus be amenable to transfer learning. Likewise, PTMs that differ in the molecular characteristics of the PTM itself, but which utilize related enzymes, such as ubiquitin E3 ligases and SUMO E3 ligases, may also be amenable to transfer learning. Finally, all datasets and code developed during this study has been made freely available to the bioinformatics community at https://github.com/dukkakc/DTLDephos to further contribute toward the study of dephosphorylation.

## Data Availability Statement

The datasets presented in this study can be found in online repositories. The names of the repository/repositories and accession number(s) can be found below: https://github.com/dukkakc/DTLDephos.

## Author Contributions

DK, RN, MK, and DC conceived, designed the experiments, and revised the manuscript. MC, HI, NT, and SC performed the experiments and data analysis. MC, DK, RN, and SC wrote the manuscript. All authors read and approved the final manuscript.

## Conflict of Interest

MK is a paid consultant for Orion Pharma. The remaining authors declare that the research was conducted in the absence of any commercial or financial relationships that could be construed as a potential conflict of interest.
