# Supplementary material for: DTL-DephosSite: Deep Transfer Learning Based Approach to Predict Dephosphorylation Sites
Source: Front Cell Dev Biol. 2021 Jun 24;9:662983. doi: 10.3389/fcell.2021.662983 (PMC8264445; doi:10.3389/fcell.2021.662983)
Supplement: Supplementary file 1 [file Data_Sheet_1.docx]

Supplementary Information

DTL-DephosSite- Deep Transfer Learning based approach to predict dephosphorylation sites

# Supplementary Figure


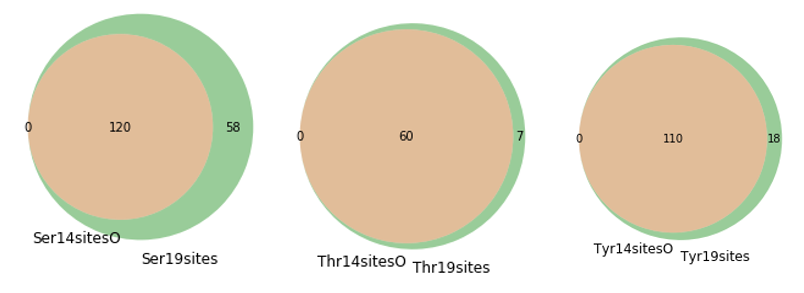


Figure S1. Venn Diagram showing increase in the Depod database from v14 to v19.

*Supplementary Tables:*

*Table S1: Summary of literature sources used to identify dephosphorylated sites for the “Downreg dataset”. The citation, number of down-regulated sites.*

| Citation | No. of dephosphorylated sites added | Detection Criteria |
| --- | --- | --- |
| Rusin SF, et al. “Quantitative Phosphoproteomics Reveals New Roles for the Protein Phosphatase Pp6 in Mitotic Cells.” *Science Signaling*, vol. 8, no. 398, 2015, p. 12., doi:10.1126/scisignal.aab3138.[1] | 298 phosphopeptides on 220 proteins | To assess significance of enrichment of terms, a hypergeometric test and Benjamini & Hochberg false discovery rate (FDR) correction were used. For a processes or component to be considered as “enriched,” a corrected P-value cutoff of 0.05 was applied. |
| Sarhan AR, et al. “Quantitative Phosphoproteomics Reveals a Role for Collapsin Response Mediator Protein 2 in Pdgf-Induced Cell Migration.” *Scientific Reports*, vol. 7, no. 1, 2017, pp. 3970–3970., doi:10.1038/s41598-017-04015-x. [2] | 45 down regulated sites | Ratio L+PDGF/M-PDGF values < 0.6 provides the magnitude and significance of differential phosphopeptide abundance in PDGF-stimulated versus unstimulated cells. Phosphopeptides are deemed significantly up- or down-regulated if they have an adjusted p value < 0.05 and exhibit a >1.5 fold-change in abundance. |
| Rusin, Scott F, et al. “Identification of Candidate Casein Kinase 2 Substrates in Mitosis by Quantitative Phosphoproteomics.” *Frontiers in Cell and Developmental Biology*, vol. 5, 2017, doi:10.3389/fcell.2017.00097.[3] | 330 phosphorylation sites on 202 proteins as significantly decreased in abundance upon inhibition of CK2 activity. | In total 4,659 phosphopeptides were quantified with a p < 0.05, using Student’s t test, shown in their supplementary Data of phosphoproteomics analysis of CX-4945 treated, mitotically-arrested HeLa cells. Log ratio < -1 was taken as criteria to determine down regulation of phosphosites. |
| Kao L, et al. “Global Analysis of Cdc14 Dephosphorylation Sites Reveals Essential Regulatory Role in Mitosis and Cytokinesis.” *Molecular & Cellular Proteomics : Mcp*, vol. 13, no. 2, 2014, pp. 594–605., doi:10.1074/mcp.M113.032680.[4] | Quantitative mass spectrometry identified a total of 835 dephosphorylation sites on 455 potential Cdc14 substrates in vivo. | As tabulated in Supplementary Table mcp.M113.032680-7.xls in Kao et al, a site was considered enriched if log_2_(cdc14-1/CDC14) > 1 and reduced if log_2_(cdc14-1/CDC14) < 1 phosphoproteins in this study |

Table S2: Percentage increase in dataset

DEPOD-19 ComDephos Fold Increase

ST 191 1112 4.82

Y 1011 25 0.24

Total 292 1237 3.24

*Table S3: Phosphorylation dataset Creation for transfer learning: Extracted through windows from the Musite Phos dataset, without any ‘-‘.*

| Phosphorylation-ST | Positive | Negative |
| --- | --- | --- |
| Train | 30095 | 64127 |
| Test | 1849 | 3912 |

*Table S4: Independent test results on feature based RF model on Dephos datasets on their respective ST residue for window size 33. Features used are K-spaced Amino Acid Pairs(KAAP), High Quality Indices(HQI) and Accumulative Hydrophobicity(ACH). MCC: Matthew’s Correlation Coefficient; SN: sensitivity, SP: Specificity; ACC:Accuracy; ROC:* area under the ROC curve

| Dataset | Residue | MCC | SP | SN | ACC | ROC_AUC |
| --- | --- | --- | --- | --- | --- | --- |
| Depod-19 | ST | 0.41 | 0.73 | 0.67 | 0.70 | 0.83 |
|  | Y | 0.35 | 0.65 | 0.70 | 0.67 | 0.77 |
| ComDephos | ST | 0.48 | 0.72 | 0.76 | 0.74 | 0.80 |
|  | Y | 0.16 | 0.65 | 0.50 | 0.58 | 0.68 |

*Table S5: Independent test results on Bi-LSTM model with different dephosphorylation datasets for different window sizes for ST residue* *MCC: Matthew’s Correlation Coefficient; SN: sensitivity, SP: Specificity; ROC_AUC:* area under the ROC curve

| Win | DEPOD19 | | | | ComDephos | | | |
| --- | --- | --- | --- | --- | --- | --- | --- | --- |
|  | MCC | SP | SN | ROC_AUC | MCC | SP | SN | ROC_AUC |
| 23 | 0.29 | 0.51 | 0.77 | 0.71 | 0.45 | 0.64 | 0.81 | 0.81 |
| 25 | 0.32 | 0.54 | 0.77 | 0.71 | 0.40 | 0.71 | 0.68 | 0.77 |
| 27 | 0.34 | 0.62 | 0.72 | 0.76 | 0.43 | 0.69 | 0.73 | 0.78 |
| 29 | 0.36 | 0.69 | 0.67 | 0.75 | 0.40 | 0.6 | 0.79 | 0.79 |
| 31 | 0.36 | 0.49 | 0.85 | 0.79 | 0.46 | 0.71 | 0.76 | 0.81 |
| 33 | 0.34 | 0.79 | 0.54 | 0.74 | 0.43 | 0.56 | 0.85 | 0.80 |

*Table S6: Independent test results on Bi-LSTM model on phosphorylation dataset for different window sizes for Y residue, obtaining window-specific Phos-Model* *MCC: Matthew’s Correlation Coefficient; SN: sensitivity; SP: Specificity; ROC_AUC: area under the ROC curve*

| Win size | MCC | SP | SN | ROC_AUC |
| --- | --- | --- | --- | --- |
| 23 | 0.58 | 0.78 | 0.8 | 0.87 |
| 25 | 0.58 | 0.73 | 0.84 | 0.87 |
| 27 | 0.59 | 0.78 | 0.81 | 0.88 |
| 29 | 0.6 | 0.78 | 0.81 | 0.88 |
| 31 | 0.59 | 0.76 | 0.83 | 0.88 |
| 33 | 0.59 | 0.76 | 0.83 | 0.88 |

*Table S7. 5-fold cross-validation of various window sizes using Depod19 dataset for ST residue. MCC: Matthew’s Correlation Coefficient; SD: Standard Deviation; SN: sensitivity, SP: Specificity; SD: Standard Deviation; ACC: Accuracy; ROC_AUC: area under the ROC curve*

| Win | MCC ± SD | SP ± SD | SN ± SD | ACC ± SD | ROC_AUC |
| --- | --- | --- | --- | --- | --- |
| 23 | 0.67 ± 0.07 | 0.87 ± 0.04 | 0.80 ±0.05 | 0.83 ±0.03 | 0.89±0.04 |
| 25 | 0.68 ± 0.11 | 0.85 ± 0.07 | 0.84 ±0.04 | 0.84 ±0.05 | 0.89 ±0.04 |
| 27 | 0.66 ± 0.07 | 0.83 ± 0.05 | 0.82 ± 0.03 | 0.83 ±0.03 | 0.88 ±0.02 |
| 29 | 0.71 ± 0.07 | **0.87 ± 0.05** | 0.84 ±0.04 | 0.85 ±0.03 | **0.91** ±0.03 |
| 31 | **0.70 ± 0.06** | 0.85 ± 0.05 | 0.85 ± 0.03 | 0.85 ±0.03 | 0.89 ±0.02 |
| 33 | **0.72 ± 0.08** | 0.83 ± 0.08 | **0.89 ± 0.02** | **0.86 ±0.04** | 0.89 ±0.02 |

*Table S8. Selection of window size for Depod19 dataset on Y residue MCC: Matthew’s Correlation Coefficient; SD: Standard Deviation; SN: sensitivity, SP: Specificity; ACC: Accuracy; ROC_AUC: area under the ROC curve*

| Win | MCC ± SD | SP ± SD | SN ± SD | Accuracy ± SD | ROC_AUC |
| --- | --- | --- | --- | --- | --- |
| 23 | 0.48 ± 0.19 | 0.68 ±0.12 | 0.79 ±0.12 | 0.74 ±0.10 | 0.77 |
| 25 | 0.49 ± 0.14 | 0.71 ±0.15 | 0.75 ±0.08 | 0.74 ±0.06 | 0.75 |
| 27 | 0.45 ± 0.19 | **0.75 ±0.14** | 0.69 ±0.11 | 0.73 ±0.09 | 0.75 |
| 29 | 0.51 ± 0.17 | 0.72 ±0.07 | 0.78 ±0.07 | 0.75 ±0.09 | **0.78** |
| 31 | **0.52 ± 0.10** | 0.72 ±0.09 | **0.80 ±0.04** | **0.76 ±0.05** | **0.78** |
| 33 | 0.48 ± 0.19 | 0.68 ±0.12 | 0.79 ±0.12 | 0.74 ±0.10 | 0.77 |

Table S9: Comparison of machine learning models on the Independent test set. *MCC: Matthew’s Correlation Coefficient; SN: sensitivity, SP: Specificity; ACC: Accuracy*

|  | MCC | SN | SP | ACC |
| --- | --- | --- | --- | --- |
| RF | 0.48 | 0.73 | 0.75 | 0.74 |
| XGBoost | 0.51 | 0.73 | 0.78 | 0.75 |

Creation of Negative Set:

We have divided the negative dataset into three sets and evaluated the model using each negative dataset:

1. Negative Set 1: Phos minus Dephos (phos – dephos): this set provides information about experimentally verified phosphorylation sites that are not known to be dephosphorylated (it is expected to be small).
2. Negative Set 2: All STY sites minus Phos (all - phos): This set provides information about sites that are not known to be phosphorylated (i.e., sites without any phosphorylation-related annotation). The “allset” was composed of all S,T, and Y residues found in each protein in the dephosphorylation set(positive set). The phosphorylation sites in these proteins were then removed from this set to form Negative set 2. We ensured there is no common sequence from the positive set.
3. Negative Set 3: All STY sites minus Dephos (all – dephos): This set provides information about sites that are not known to be dephosphorylated. The set was constructed by removing only the dephosphorylated sites from all S,T,Y sites for the respective proteins.

For the Phos and Dephos sets that were to be subtracted, we used the proteins that formed our positive set (i.e., those proteins that are known to be dephosphorylated) from our combined dephosphorylation set (ComDephos). This set is composed of a total of 624 proteins.

Next, for these 624 proteins, we created the following sets:

1. All_S, All_T, All_Y, which consists of positions for all S, T, and Y residues in the proteins, respectively.

2. Phos_S, Phos_T and Phos_Y, which consists of the phosphorylated sites in the proteins provided by the Phospho.ELM database.

3. Dephos_S, Dephos_T, Dephos_Y are the dephosphorylated sites (i.e., our positive set from the ComDephos dataset).

All these sets were defined with respect to the proteins so that the construction of the negative sets was obtained by subtraction with respect to each protein (Table R3).

*Table S10 The negative sets created by taking the positive set from the ComDephos set.*

| **Negative sites** | **ST(Train/Test)** | **Y(Train/Test)** |
| --- | --- | --- |
| Set1_phos - dephos | 4906 (3933/973) | 199 (161/38) |
| Set2_all - phos - dephos | 61,334 (49,116/12,218) | 1997 (1608/389) |
| Set3_all - dephos | 66011 (52,875/13,136) | 2187 (1752/435) |
| pos_sites | 1112(892/220) | 125(100/25) |

Next, the positive and negative sets were run through duplicacy removal to ensure that no redundant sequences were shared between the positive and negative sets. The dataset was then divided into training and test sets using an 80:20 ratio. We ensured that there were no common or duplicate sequences in the training and test sets. The Bi-LSTM model was then trained on the training set and tested on the independent test set. The results are tabulated in Table R4). These analyses suggest that negative Set 2 (i.e., all - phos - dephos) outperforms the others.

*Table S10. Independent Test Results on ComDephos using each new negative dataset. MCC: Matthew’s Correlation Coefficient; SN: sensitivity, SP: Specificity; ACC: Accuracy; ROC_AUC: area under the ROC curve*

|  |  | MCC | Specificity | Sensitivity | Accuracy | ROC_AUC |
| --- | --- | --- | --- | --- | --- | --- |
| Set1 | ST | 0.11 | 0.68 | 0.43 | 0.55 | 0.58 |
|  | ST_transphos | 0.24 | 0.36 | 0.85 | 0.60 | 0.67 |
|  | Y_transSTde | 0.22 | 0.28 | 0.89 | 0.59 | 0.67 |
| Set2 | ST | 0.32 | 0.68 | 0.64 | 0.66 | 0.74 |
|  | **ST_transphos** | 0.63 | 0.77 | 0.86 | 0.81 | **0.90** |
|  | Y_transSTde | 0.36 | 0.53 | 0.82 | 0.67 | 0.74 |
| Set3 | ST | 0.35 | 0.66 | 0.69 | 0.68 | 0.73 |
|  | ST_transphos | 0.68 | 0.84 | 0.84 | 0.84 | **0.90** |
|  | Y_transSTde | 0.64 | 0.88 | 0.75 | 0.82 | 0.89 |

Architecture of models used for Comparison in Table 5

Architecture descriptions for each of the models is provided below:

DeepPhos Model[5]: A Densely Connected Convolutional Neural Network (DC-CNN)-based model, which employs three DC-CNN blocks, each taking window sizes of 15, 33 and 51. The DC-CNN blocks utilized a 1D convolution along the protein sequence length. The model architecture was used from the DeepPhos github.

CNN Model: The CNN model employed three 2D convolutional layers, one maxpooling layer, a fully connected layer with two dense layers, and an output layer. The input sequences were integer-encoded to the input embedding layer using a window size of 33. The first convolutional layer had 64 filters with kernel size of (17,3) followed by a drop-out layer and maxpooling 2D layer. Similarly, the other two blocks of convolutional layers were composed of 128 and 256 filters, respectively, with a kernel size of (3,3). These were followed by two dense layers of 768 and 256 neurons, each followed by a drop-out layer at drop-out rate of 0.5. Finally, a softmax layer was used to provide the predictions.

LSTM: Similar to the CNN architecture, the window sequences of the LSTM model were integer encoded and provided to the Embedding input layer. The model used two LSTM layers of 128 neurons and 64 neurons. A recurrent drop-out at rate of 0.4 was applied to the second LSTM layer. This was followed by two dense layers of 128 and 64 neurons. The drop-out layers were applied after each dense layer at a drop-out rate of 0.4. Finally, a softmax layer was used to provide the predictions.

Supplementary Table S12

Supplemental DownRegulation Dataset mined from literature

| sp\|A0MZ66\|SHOT1_HUMAN | 467 | RSLKSLDPE | S |
| --- | --- | --- | --- |
| sp\|A1L170\|CA226_HUMAN | 223 | RRASSPSLI | S |
| sp\|A2AHC3\|CAMP1_MOUSE | 560 | MPRTSPQAP | S |
| sp\|A7KAX9\|RHG32_HUMAN | 587 | SRPKSLLVS | S |
| sp\|A7KAX9\|RHG32_HUMAN | 1820 | AKAISPEGE | S |
| sp\|F8VPU2\|FARP1_MOUSE | 427 | GPHQSPALP | S |
| sp\|O00159\|MYO1C_HUMAN | 139 | AVMISGESG | S |
| sp\|O00159\|MYO1C_HUMAN | 142 | ISGESGAGK | S |
| sp\|O00159\|MYO1C_HUMAN | 147 | GAGKTEATK | T |
| sp\|O00193\|SMAP_HUMAN | 87 | QYQQSMDSK | S |
| sp\|O00203\|AP3B1_HUMAN | 276 | NFYESDDDQ | S |
| sp\|O00203\|AP3B1_HUMAN | 274 | GKNFYESDD | Y |
| sp\|O00299\|CLIC1_HUMAN | 146 | NYLTSPLPE | S |
| sp\|O00429\|DNM1L_HUMAN | 616 | IMPASPQKG | S |
| sp\|O00470\|MEIS1_HUMAN | 196 | GGSKSDSED | S |
| sp\|O00470\|MEIS1_HUMAN | 194 | REGGSKSDS | S |
| sp\|O00567\|NOP56_HUMAN | 519 | EELMSSDLE | S |
| sp\|O00567\|NOP56_HUMAN | 511 | KKKKSFSKE | S |
| sp\|O00567\|NOP56_HUMAN | 520 | ELMSSDLEE | S |
| sp\|O00767\|ACOD_HUMAN | 198 | KEKGSTLDL | S |
| sp\|O00767\|ACOD_HUMAN | 199 | EKGSTLDLS | T |
| sp\|O08553\|DPYL2_MOUSE | 514 | PKTVTPASS | T |
| sp\|O14523\|C2C2L_HUMAN | 662 | GLSQSHDDL | S |
| sp\|O14639\|ABLM1_HUMAN | 587 | KRRSSGREE | S |
| sp\|O14647\|CHD2_HUMAN | 130 | EEASSGSES | S |
| sp\|O14974\|MYPT1_HUMAN | 507 | RRLASTSDI | S |
| sp\|O15013\|ARHGA_HUMAN | 1284 | DLSSSSGSL | S |
| sp\|O15013\|ARHGA_HUMAN | 1285 | LSSSSGSLS | S |
| sp\|O15117\|FYB1_HUMAN | 225 | SSKGSPAPL | S |
| sp\|O15155\|BET1_HUMAN | 50 | IKSLSIEIG | S |
| sp\|O15164\|TIF1A_HUMAN | 1028 | FSDDSDDDF | S |
| sp\|O15164\|TIF1A_HUMAN | 1025 | DNKFSDDSD | S |
| sp\|O15173\|PGRC2_HUMAN | 90 | AGEESPATS | S |
| sp\|O15231\|ZN185_HUMAN | 453 | ERQSSPSGS | S |
| sp\|O15446\|RPA34_HUMAN | 285 | DTVLSPTKK | S |
| sp\|O43149\|ZZEF1_HUMAN | 1518 | EEPLSPSTP | S |
| sp\|O43149\|ZZEF1_HUMAN | 1509 | AADVSPATA | S |
| sp\|O43149\|ZZEF1_HUMAN | 1521 | LSPSTPTRR | T |
| sp\|O43159\|RRP8_HUMAN | 106 | PCSDSEEEV | S |
| sp\|O43159\|RRP8_HUMAN | 124 | ALVGSDSAE | S |
| sp\|O43182\|RHG06_HUMAN | 754 | GMTGSSGDI | S |
| sp\|O43294\|TGFI1_HUMAN | 141 | SLPSSPSPG | S |
| sp\|O43379\|WDR62_HUMAN | 1144 | TGYASPDRT | S |
| sp\|O43426\|SYNJ1_HUMAN | 1391 | ENMRSSPNP | S |
| sp\|O43432\|IF4G3_HUMAN | 495 | NSRRSPVPA | S |
| sp\|O43432\|IF4G3_HUMAN | 232 | LKSPSPVLR | S |
| sp\|O43516\|WIPF1_HUMAN | 398 | ALPATPQLP | T |
| sp\|O43561\|LAT_HUMAN | 84 | PIPRSPQPL | S |
| sp\|O43583\|DENR_HUMAN | 73 | TVENSPKQE | S |
| sp\|O43639\|NCK2_HUMAN | 74 | NLKDTLGLG | T |
| sp\|O43823\|AKAP8_HUMAN | 328 | EGDFSENDD | S |
| sp\|O54774\|AP3D1_MOUSE | 760 | LPTESDEDI | S |
| sp\|O54774\|AP3D1_MOUSE | 758 | SSLPTESDE | T |
| sp\|O60271\|JIP4_HUMAN | 363 | GYKGSSTPT | S |
| sp\|O60271\|JIP4_HUMAN | 595 | KRSSTLSQL | T |
| sp\|O60271\|JIP4_HUMAN | 217 | DGLLTPDAQ | T |
| sp\|O60341\|KDM1A_HUMAN | 135 | SEDEYYSEE | Y |
| sp\|O60341\|KDM1A_HUMAN | 136 | EDEYYSEEE | Y |
| sp\|O60343\|TBCD4_HUMAN | 550 | ENATSSGRF | S |
| sp\|O60343\|TBCD4_HUMAN | 551 | NATSSGRFK | S |
| sp\|O60381\|HBP1_HUMAN | 380 | QRRASLSCG | S |
| sp\|O60675\|MAFK_HUMAN | 25 | APVLSDDEL | S |
| sp\|O60749\|SNX2_HUMAN | 119 | SKSMSAPVI | S |
| sp\|O60825\|F262_HUMAN | 466 | MRRNSFTPL | S |
| sp\|O60825\|F262_HUMAN | 473 | PLSSSNTIR | S |
| sp\|O60825\|F262_HUMAN | 175 | VKVSSPDYP | S |
| sp\|O60825\|F262_HUMAN | 475 | SSSNTIRRP | T |
| sp\|O60832\|DKC1_HUMAN | 455 | SESESDETP | S |
| sp\|O60832\|DKC1_HUMAN | 451 | RKRESESES | S |
| sp\|O60832\|DKC1_HUMAN | 458 | ESDETPPAA | T |
| sp\|O60832\|DKC1_HUMAN | 453 | RESESESDE | S |
| sp\|O60841\|IF2P_HUMAN | 186 | SSGESGDES | S |
| sp\|O60841\|IF2P_HUMAN | 588 | SKEMSSDSE | S |
| sp\|O60841\|IF2P_HUMAN | 137 | MYSGSDDDD | S |
| sp\|O60841\|IF2P_HUMAN | 134 | KVEMYSGSD | Y |
| sp\|O60841\|IF2P_HUMAN | 135 | VEMYSGSDD | S |
| sp\|O60841\|IF2P_HUMAN | 214 | PNIESGNED | S |
| sp\|O60890\|OPHN1_HUMAN | 703 | TKTPSFHIK | S |
| sp\|O70318\|E41L2_MOUSE | 666 | PQASSHETL | S |
| sp\|O75069\|TMCC2_HUMAN | 438 | NKFGSADNI | S |
| sp\|O75122\|CLAP2_HUMAN | 1025 | PYNYSDSIS | S |
| sp\|O75122\|CLAP2_HUMAN | 1029 | SDSISPFNK | S |
| sp\|O75146\|HIP1R_HUMAN | 1045 | AQKPSVAPR | S |
| sp\|O75152\|ZC11A_HUMAN | 290 | KRKFSAGGD | S |
| sp\|O75179\|ANR17_HUMAN | 2044 | VSSPSSPSP | S |
| sp\|O75190\|DNJB6_HUMAN | 277 | PHCLSEEEG | S |
| sp\|O75391\|SPAG7_HUMAN | 114 | EFAPSDEEL | S |
| sp\|O75396\|SC22B_HUMAN | 137 | RNLGSINTE | S |
| sp\|O75533\|SF3B1_HUMAN | 129 | TMIISPERL | S |
| sp\|O75962\|TRIO_HUMAN | 1633 | GDGSSQPDT | S |
| sp\|O75962\|TRIO_HUMAN | 1632 | QGDGSSQPD | S |
| sp\|O76021\|RL1D1_HUMAN | 361 | ATNESEDEI | S |
| sp\|O76021\|RL1D1_HUMAN | 358 | QVKATNESE | T |
| sp\|O76094\|SRP72_HUMAN | 616 | ELDASKTVS | S |
| sp\|O76094\|SRP72_HUMAN | 610 | TAGASSELD | S |
| sp\|O94827\|PKHG5_HUMAN | 249 | SRRESLDIL | S |
| sp\|O94868\|FCSD2_HUMAN | 681 | YFPRSPSAN | S |
| sp\|O94876\|TMCC1_HUMAN | 414 | NFQSSPKYG | S |
| sp\|O94880\|PHF14_HUMAN | 302 | SNEDSLILE | S |
| sp\|O94885\|SASH1_HUMAN | 743 | TRKASLLSA | S |
| sp\|O94915\|FRYL_HUMAN | 1978 | ARTRSLSSL | S |
| sp\|O94992\|HEXI1_HUMAN | 237 | SDDTSDDDF | S |
| sp\|O95218\|ZRAB2_HUMAN | 120 | EREESDGEY | S |
| sp\|O95218\|ZRAB2_HUMAN | 114 | ENVEYIERE | Y |
| sp\|O95235\|KI20A_HUMAN | 48 | SVVSTSLED | T |
| sp\|O95239\|KIF4A_HUMAN | 803 | TFSLTEVRG | T |
| sp\|O95239\|KIF4A_HUMAN | 801 | RRTFSLTEV | S |
| sp\|O95425\|SVIL_HUMAN | 769 | ARLPSPTVA | S |
| sp\|O95456\|PSMG1_HUMAN | 18 | CRAGTEDEE | T |
| sp\|O95644\|NFAC1_HUMAN | 233 | TLLGSPRHS | S |
| sp\|O95671\|ASML_HUMAN | 239 | FEDLSDVEG | S |
| sp\|O95671\|ASML_HUMAN | 234 | PAADTFEDL | T |
| sp\|O95674\|CDS2_HUMAN | 33 | GETASDSES | S |
| sp\|O95785\|WIZ_HUMAN | 1155 | SPTASPPPT | S |
| sp\|O95785\|WIZ_HUMAN | 996 | ALAGSPTPK | S |
| sp\|O95785\|WIZ_HUMAN | 1151 | PLGHSPTAS | S |
| sp\|O95785\|WIZ_HUMAN | 1146 | PPPGSPLGH | S |
| sp\|O95785\|WIZ_HUMAN | 989 | LLKKTPLAL | T |
| sp\|O95785\|WIZ_HUMAN | 1153 | GHSPTASPP | T |
| sp\|O95810\|CAVN2_HUMAN | 370 | SGMDSNIDL | S |
| sp\|O95810\|CAVN2_HUMAN | 293 | PFKVSPLTF | S |
| sp\|O95810\|CAVN2_HUMAN | 287 | SSGKSSPFK | S |
| sp\|O95810\|CAVN2_HUMAN | 296 | VSPLTFGRK | T |
| sp\|O95817\|BAG3_HUMAN | 180 | ASDCSSSSS | S |
| sp\|O95817\|BAG3_HUMAN | 194 | SSGRSSLGS | S |
| sp\|O95817\|BAG3_HUMAN | 195 | SGRSSLGSH | S |
| sp\|O95817\|BAG3_HUMAN | 173 | ERSQSPAAS | S |
| sp\|O95819\|M4K4_HUMAN | 631 | TTSRSPVLS | S |
| sp\|O95835\|LATS1_HUMAN | 464 | VRSNSFNNP | S |
| sp\|P02545\|LMNA_HUMAN | 51 | DRVRSLETE | S |
| sp\|P04920\|B3A2_HUMAN | 172 | RTSPSSPAP | S |
| sp\|P04920\|B3A2_HUMAN | 173 | TSPSSPAPL | S |
| sp\|P05204\|HMGN2_HUMAN | 25 | PQRRSARLS | S |
| sp\|P05204\|HMGN2_HUMAN | 29 | SARLSAKPA | S |
| sp\|P05387\|RLA2_HUMAN | 86 | PAAGSAPAA | S |
| sp\|P05455\|LA_HUMAN | 366 | TKFASDDEH | S |
| sp\|P06493\|CDK1_HUMAN | 19 | YGVVYKGRH | Y |
| sp\|P07199\|CENPB_HUMAN | 307 | SLDTSGLRH | S |
| sp\|P07199\|CENPB_HUMAN | 306 | QSLDTSGLR | T |
| sp\|P07900\|HS90A_HUMAN | 263 | EDVGSDEEE | S |
| sp\|P07948\|LYN_HUMAN | 316 | EEPIYIITE | Y |
| sp\|P08559\|ODPA_HUMAN | 300 | DPGVSYRTR | S |
| sp\|P08559\|ODPA_HUMAN | 289 | QTYRYHGHS | Y |
| sp\|P08559\|ODPA_HUMAN | 232 | GMGTSVERA | S |
| sp\|P08567\|PLEK_HUMAN | 117 | STRRSIRLP | S |
| sp\|P08572\|CO4A2_HUMAN | 467 | GLPGSPGAR | S |
| sp\|P08581\|MET_HUMAN | 966 | KDLGSELVR | S |
| sp\|P08670\|VIME_HUMAN | 56 | LYASSPGGV | S |
| sp\|P08670\|VIME_HUMAN | 65 | YATRSSAVR | S |
| sp\|P08670\|VIME_HUMAN | 66 | ATRSSAVRL | S |
| sp\|P08670\|VIME_HUMAN | 63 | GVYATRSSA | T |
| sp\|P08670\|VIME_HUMAN | 61 | PGGVYATRS | Y |
| sp\|P08670\|VIME_HUMAN | 51 | STSRSLYAS | S |
| sp\|P09874\|PARP1_HUMAN | 277 | PSGESAILD | S |
| sp\|P10071\|GLI3_HUMAN | 865 | SRRSSGISP | S |
| sp\|P11388\|TOP2A_HUMAN | 1377 | KSVVSDLEA | S |
| sp\|P12270\|TPR_HUMAN | 379 | LAAMSPTAA | S |
| sp\|P12270\|TPR_HUMAN | 1838 | EEEDSTIEA | S |
| sp\|P12956\|XRCC6_HUMAN | 560 | KVEYSEEEL | S |
| sp\|P13639\|EF2_HUMAN | 59 | RFTDTRKDE | T |
| sp\|P13639\|EF2_HUMAN | 54 | RAGETRFTD | T |
| sp\|P13807\|GYS1_HUMAN | 645 | SVPPSPSLS | S |
| sp\|P13807\|GYS1_HUMAN | 649 | SPSLSRHSS | S |
| sp\|P13807\|GYS1_HUMAN | 636 | QGYRYPRPA | Y |
| sp\|P13861\|KAP2_HUMAN | 78 | AKGDSESEE | S |
| sp\|P13861\|KAP2_HUMAN | 58 | TPRQSLGHP | S |
| sp\|P14873\|MAP1B_MOUSE | 1293 | ARSVSPGVT | S |
| sp\|P15408\|FOSL2_HUMAN | 215 | SGGGSVGAV | S |
| sp\|P15408\|FOSL2_HUMAN | 230 | LEEDSPSSS | S |
| sp\|P16546\|SPTN1_MOUSE | 1031 | AQSASRENL | S |
| sp\|P16949\|STMN1_HUMAN | 25 | ELILSPRSK | S |
| sp\|P18206\|VINC_HUMAN | 434 | DILRSLGEI | S |
| sp\|P18583\|SON_HUMAN | 949 | GQDPYRLGH | Y |
| sp\|P18583\|SON_HUMAN | 39 | GETNTPIEG | T |
| sp\|P18858\|DNLI1_HUMAN | 141 | LEEQSEDED | S |
| sp\|P19634\|SL9A1_HUMAN | 616 | KSLPSERIL | S |
| sp\|P19634\|SL9A1_HUMAN | 605 | VSTVSMQNI | S |
| sp\|P20810\|ICAL_HUMAN | 133 | KEKKSLTPA | S |
| sp\|P20810\|ICAL_HUMAN | 561 | PRDTSQSDK | S |
| sp\|P20810\|ICAL_HUMAN | 135 | KKSLTPAVP | T |
| sp\|P22626\|ROA2_HUMAN | 212 | GFGDSRGGG | S |
| sp\|P23528\|COF1_HUMAN | 129 | KKKLTGIKH | T |
| sp\|P25490\|TYY1_HUMAN | 378 | VRIHTGDRP | T |
| sp\|P26358\|DNMT1_HUMAN | 714 | PEMPSPKKM | S |
| sp\|P27816\|MAP4_HUMAN | 1000 | SKKVSYSHI | S |
| sp\|P27816\|MAP4_HUMAN | 521 | GKDVTPPPE | T |
| sp\|P27987\|IP3KB_HUMAN | 264 | GIPASPRCG | S |
| sp\|P27987\|IP3KB_HUMAN | 269 | PRCGSPTAM | S |
| sp\|P27987\|IP3KB_HUMAN | 166 | QAPRSPRLG | S |
| sp\|P28290\|ITPI2_HUMAN | 1040 | VRMPSPFRS | S |
| sp\|P28290\|ITPI2_HUMAN | 593 | RKSGSQDFP | S |
| sp\|P29474\|NOS3_HUMAN | 1177 | IRTQSFSLQ | S |
| sp\|P29474\|NOS3_HUMAN | 1175 | SRIRTQSFS | T |
| sp\|P29474\|NOS3_HUMAN | 615 | IRFNSISCS | S |
| sp\|P29692\|EF1D_HUMAN | 162 | DLFGSDNEE | S |
| sp\|P30414\|NKTR_HUMAN | 463 | ILIPSDIES | S |
| sp\|P30622\|CLIP1_HUMAN | 48 | STPSSETQE | S |
| sp\|P30622\|CLIP1_HUMAN | 45 | KASSTPSSE | T |
| sp\|P35221\|CTNA1_HUMAN | 658 | TSVQTEDDQ | T |
| sp\|P35221\|CTNA1_HUMAN | 654 | VRSRTSVQT | T |
| sp\|P35221\|CTNA1_HUMAN | 655 | RSRTSVQTE | S |
| sp\|P35251\|RFC1_HUMAN | 71 | YDSDSESEE | S |
| sp\|P35251\|RFC1_HUMAN | 69 | IIYDSDSES | S |
| sp\|P35283\|RAB12_MOUSE | 20 | LGAVSPALS | S |
| sp\|P35367\|HRH1_HUMAN | 380 | LRSGSNTGL | S |
| sp\|P35579\|MYH9_HUMAN | 1943 | AGDGSDEEV | S |
| sp\|P35613\|BASI_HUMAN | 362 | DDAGSAPLK | S |
| sp\|P36915\|GNL1_HUMAN | 48 | REEQTDTSD | T |
| sp\|P36915\|GNL1_HUMAN | 50 | EQTDTSDGE | T |
| sp\|P36915\|GNL1_HUMAN | 51 | QTDTSDGES | S |
| sp\|P38432\|COIL_HUMAN | 122 | EGEETEPDC | T |
| sp\|P39880\|CUX1_HUMAN | 1270 | KPYPSPKTI | S |
| sp\|P40818\|UBP8_HUMAN | 378 | PLNISTPVE | S |
| sp\|P41091\|IF2G_HUMAN | 108 | SCGSSTPDE | S |
| sp\|P41236\|IPP2_HUMAN | 121 | QEQESSGEE | S |
| sp\|P41236\|IPP2_HUMAN | 122 | EQESSGEED | S |
| sp\|P42331\|RHG25_HUMAN | 487 | RRTMSQDLR | S |
| sp\|P42331\|RHG25_HUMAN | 485 | GHRRTMSQD | T |
| sp\|P42566\|EPS15_HUMAN | 563 | PARSSPELL | S |
| sp\|P42566\|EPS15_HUMAN | 796 | NKLDSPDPF | S |
| sp\|P42768\|WASP_HUMAN | 291 | SKLIYDFIE | Y |
| sp\|P43121\|MUC18_HUMAN | 628 | LQGSSGDKR | S |
| sp\|P43243\|MATR3_HUMAN | 150 | KRRRTEEGP | T |
| sp\|P46013\|KI67_HUMAN | 2638 | KEPASGDEG | S |
| sp\|P46013\|KI67_HUMAN | 543 | LVMHTPPVL | T |
| sp\|P46013\|KI67_HUMAN | 2625 | RLTQTSGQS | T |
| sp\|P46100\|ATRX_HUMAN | 1061 | KDELSDYAE | S |
| sp\|P46821\|MAP1B_HUMAN | 1917 | TKSPSDSGY | S |
| sp\|P46938\|YAP1_MOUSE | 90 | PKSHSRQAS | S |
| sp\|P49589\|SYCC_HUMAN | 307 | DLSISADRL | S |
| sp\|P49790\|NU153_HUMAN | 522 | SSTGSPMFK | S |
| sp\|P49790\|NU153_HUMAN | 516 | VQMTSPSST | S |
| sp\|P49792\|RBP2_HUMAN | 799 | YSPKTPPRW | T |
| sp\|P49792\|RBP2_HUMAN | 795 | KSYKYSPKT | Y |
| sp\|P50552\|VASP_HUMAN | 315 | EKNSTTLPR | T |
| sp\|P50552\|VASP_HUMAN | 239 | LRKVSKQEE | S |
| sp\|P50552\|VASP_HUMAN | 316 | KNSTTLPRM | T |
| sp\|P51991\|ROA3_HUMAN | 356 | GGRSSGSPY | S |
| sp\|P51991\|ROA3_HUMAN | 358 | RSSGSPYGG | S |
| sp\|P52272\|HNRPM_HUMAN | 528 | RMGLSMERM | S |
| sp\|P52272\|HNRPM_HUMAN | 633 | NFGGSFAGS | S |
| sp\|P52272\|HNRPM_HUMAN | 637 | SFAGSFGGA | S |
| sp\|P52565\|GDIR1_HUMAN | 34 | PAQKSIQEI | S |
| sp\|P52594\|AGFG1_HUMAN | 181 | TPSQSPVVG | S |
| sp\|P52701\|MSH6_HUMAN | 18 | SPALSDANK | S |
| sp\|P52732\|KIF11_HUMAN | 39 | ASAHSIVEC | S |
| sp\|P52756\|RBM5_HUMAN | 78 | DGYHSDGDY | S |
| sp\|P53367\|ARFP1_HUMAN | 132 | VRKWSLNTY | S |
| sp\|P53814\|SMTN_HUMAN | 379 | ASSSSGSSS | S |
| sp\|P53814\|SMTN_HUMAN | 376 | TTPASSSSG | S |
| sp\|P55010\|IF5_HUMAN | 389 | AEEESSGGE | S |
| sp\|P55010\|IF5_HUMAN | 390 | EEESSGGEE | S |
| sp\|P62834\|RAP1A_HUMAN | 39 | TIEDSYRKQ | S |
| sp\|P62987\|RL40_HUMAN | 57 | GRTLSDYNI | S |
| sp\|P67809\|YBOX1_HUMAN | 165 | NYQNSESGE | S |
| sp\|P78536\|ADA17_HUMAN | 735 | PAPQTPGRL | T |
| sp\|P78559\|MAP1A_HUMAN | 1818 | PGQESPIPD | S |
| sp\|P78559\|MAP1A_HUMAN | 1776 | WLAESPVGL | S |
| sp\|P80723\|BASP1_HUMAN | 205 | GPAASAEEP | S |
| sp\|P82979\|SARNP_HUMAN | 130 | RFGISSVPT | S |
| sp\|P85037\|FOXK1_HUMAN | 253 | SVPNSCPAS | S |
| sp\|P98171\|RHG04_HUMAN | 906 | PGPRSPKAP | S |
| sp\|P98175\|RBM10_HUMAN | 797 | RAHLSENEL | S |
| sp\|Q00534\|CDK6_HUMAN | 49 | VRVQTGEEG | T |
| sp\|Q00610\|CLH1_HUMAN | 394 | GILRTPDTI | T |
| sp\|Q00688\|FKBP3_HUMAN | 100 | KETKSEETL | S |
| sp\|Q01433\|AMPD2_HUMAN | 168 | ERQISQDVK | S |
| sp\|Q01433\|AMPD2_HUMAN | 190 | LKTDSDSDL | S |
| sp\|Q01433\|AMPD2_HUMAN | 188 | DFLKTDSDS | T |
| sp\|Q02241\|KIF23_HUMAN | 867 | QELASDGEI | S |
| sp\|Q02543\|RL18A_HUMAN | 71 | VFEKSPLRV | S |
| sp\|Q02880\|TOP2B_HUMAN | 1471 | NEEDSASVF | S |
| sp\|Q02880\|TOP2B_HUMAN | 1466 | AKFDSNEED | S |
| sp\|Q02880\|TOP2B_HUMAN | 1581 | FDQDSDVDI | S |
| sp\|Q02880\|TOP2B_HUMAN | 1457 | YSQKSEDDS | S |
| sp\|Q02880\|TOP2B_HUMAN | 1461 | SEDDSAKFD | S |
| sp\|Q04690\|NF1_MOUSE | 2517 | VGQTSPRAR | S |
| sp\|Q04721\|NOTC2_HUMAN | 1778 | EALLSEEDD | S |
| sp\|Q04721\|NOTC2_HUMAN | 2093 | RSFLSLKHT | S |
| sp\|Q04721\|NOTC2_HUMAN | 2115 | TMPTSLPNL | S |
| sp\|Q04726\|TLE3_HUMAN | 296 | SSSSTPSSK | T |
| sp\|Q04726\|TLE3_HUMAN | 312 | DKSSTPGLK | T |
| sp\|Q04726\|TLE3_HUMAN | 319 | LKSNTPTPR | T |
| sp\|Q04726\|TLE3_HUMAN | 286 | DAPTSPASV | S |
| sp\|Q05209\|PTN12_HUMAN | 509 | KVSVTPPEE | T |
| sp\|Q05209\|PTN12_HUMAN | 454 | FDGNTLLNR | T |
| sp\|Q05519\|SRS11_HUMAN | 456 | TKECSVEKG | S |
| sp\|Q05519\|SRS11_HUMAN | 434 | QGYDSEKEK | S |
| sp\|Q07157\|ZO1_HUMAN | 125 | PEPVSDNEE | S |
| sp\|Q07955\|SRSF1_HUMAN | 201 | PRSPSYGRS | S |
| sp\|Q07955\|SRSF1_HUMAN | 199 | DGPRSPSYG | S |
| sp\|Q07955\|SRSF1_HUMAN | 202 | RSPSYGRSR | Y |
| sp\|Q08495\|DEMA_HUMAN | 26 | SVPGSPSSI | S |
| sp\|Q08495\|DEMA_HUMAN | 87 | ERSLSPKST | S |
| sp\|Q08495\|DEMA_HUMAN | 226 | EDDDSGEEM | S |
| sp\|Q08495\|DEMA_HUMAN | 22 | SRDSSVPGS | S |
| sp\|Q08495\|DEMA_HUMAN | 91 | SPKSTSPPP | T |
| sp\|Q08495\|DEMA_HUMAN | 156 | SVGGSPQTK | S |
| sp\|Q08945\|SSRP1_HUMAN | 662 | ESFKSKEFV | S |
| sp\|Q08945\|SSRP1_HUMAN | 437 | GMNPSYDEY | S |
| sp\|Q08945\|SSRP1_HUMAN | 444 | EYADSDEDQ | S |
| sp\|Q08945\|SSRP1_HUMAN | 667 | KEFVSSDES | S |
| sp\|Q09472\|EP300_HUMAN | 1716 | LDDESNNQQ | S |
| sp\|Q09666\|AHNK_HUMAN | 5735 | SPEASISGS | S |
| sp\|Q09666\|AHNK_HUMAN | 3362 | NFSGSKVQT | S |
| sp\|Q09666\|AHNK_HUMAN | 5530 | GLKGSEVGF | S |
| sp\|Q09666\|AHNK_HUMAN | 135 | PRLKSEDGV | S |
| sp\|Q09666\|AHNK_HUMAN | 5841 | EVTGSDDET | S |
| sp\|Q09666\|AHNK_HUMAN | 5729 | KGGVTGSPE | T |
| sp\|Q09666\|AHNK_HUMAN | 3360 | KFNFSGSKV | S |
| sp\|Q09666\|AHNK_HUMAN | 3366 | SKVQTPEVD | T |
| sp\|Q0ZGT2\|NEXN_HUMAN | 80 | EMLASDDEE | S |
| sp\|Q12802\|AKP13_HUMAN | 1876 | ERPRSAVLL | S |
| sp\|Q12815\|TROAP_HUMAN | 213 | QALISPSGP | S |
| sp\|Q12815\|TROAP_HUMAN | 223 | FHPSTRPSF | T |
| sp\|Q12830\|BPTF_HUMAN | 1763 | TKLSTPSTG | T |
| sp\|Q12888\|TP53B_HUMAN | 1665 | MGVLSGKRK | S |
| sp\|Q12888\|TP53B_HUMAN | 1317 | HRTSSGTSL | S |
| sp\|Q12888\|TP53B_HUMAN | 518 | KLMLSTSEY | S |
| sp\|Q12888\|TP53B_HUMAN | 1660 | SPRASMGVL | S |
| sp\|Q12906\|ILF3_HUMAN | 812 | NYSGSGGRS | S |
| sp\|Q12906\|ILF3_HUMAN | 810 | KFNYSGSGG | S |
| sp\|Q13017\|RHG05_HUMAN | 968 | VFLPSPRDC | S |
| sp\|Q13017\|RHG05_HUMAN | 1217 | NPAITSDQE | T |
| sp\|Q13017\|RHG05_HUMAN | 1218 | PAITSDQEL | S |
| sp\|Q13136\|LIPA1_HUMAN | 242 | SSDGSLSHE | S |
| sp\|Q13136\|LIPA1_HUMAN | 244 | DGSLSHEED | S |
| sp\|Q13136\|LIPA1_HUMAN | 761 | GALHTVSHE | T |
| sp\|Q13136\|LIPA1_HUMAN | 763 | LHTVSHEDI | S |
| sp\|Q13185\|CBX3_HUMAN | 95 | RKSLSDSES | S |
| sp\|Q13206\|DDX10_HUMAN | 577 | QDNDTGNEE | T |
| sp\|Q13242\|SRSF9_HUMAN | 109 | PTRRSDFRV | S |
| sp\|Q13416\|ORC2_HUMAN | 284 | SPSFSAELK | S |
| sp\|Q13416\|ORC2_HUMAN | 280 | LSKVSPSFS | S |
| sp\|Q13416\|ORC2_HUMAN | 282 | KVSPSFSAE | S |
| sp\|Q13424\|SNTA1_HUMAN | 173 | MKDVSPYFK | S |
| sp\|Q13428\|TCOF_HUMAN | 111 | STNSSVLGA | S |
| sp\|Q13428\|TCOF_HUMAN | 316 | GKGATPAPP | T |
| sp\|Q13428\|TCOF_HUMAN | 310 | PAKGTPGKG | T |
| sp\|Q13435\|SF3B2_HUMAN | 302 | TDARSSLGQ | S |
| sp\|Q13435\|SF3B2_HUMAN | 343 | VRGVSSESS | S |
| sp\|Q13435\|SF3B2_HUMAN | 344 | RGVSSESSG | S |
| sp\|Q13435\|SF3B2_HUMAN | 303 | DARSSLGQS | S |
| sp\|Q13442\|HAP28_HUMAN | 60 | KSLDSDESE | S |
| sp\|Q13459\|MYO9B_HUMAN | 1354 | ERRTSFSTS | S |
| sp\|Q13459\|MYO9B_HUMAN | 766 | SLLQSLSRL | S |
| sp\|Q13459\|MYO9B_HUMAN | 1353 | EERRTSFST | T |
| sp\|Q13523\|PRP4B_HUMAN | 368 | RRSRSPLLN | S |
| sp\|Q13547\|HDAC1_HUMAN | 393 | IPEESGDED | S |
| sp\|Q13547\|HDAC1_HUMAN | 421 | EEEFSDSEE | S |
| sp\|Q13547\|HDAC1_HUMAN | 423 | EFSDSEEEG | S |
| sp\|Q13586\|STIM1_HUMAN | 257 | RAEQSLHDL | S |
| sp\|Q13586\|STIM1_HUMAN | 575 | KLPDSPALA | S |
| sp\|Q13595\|TRA2A_HUMAN | 264 | SPSPYYSRY | Y |
| sp\|Q13595\|TRA2A_HUMAN | 260 | YRRRSPSPY | S |
| sp\|Q13610\|PWP1_HUMAN | 485 | ARNSSISGP | S |
| sp\|Q13625\|ASPP2_HUMAN | 737 | KKRSSITEP | S |
| sp\|Q13813\|SPTN1_HUMAN | 1031 | AQSASRENL | S |
| sp\|Q14151\|SAFB2_HUMAN | 507 | EKLSSVDRH | S |
| sp\|Q14151\|SAFB2_HUMAN | 513 | DRHHSVEIK | S |
| sp\|Q14157\|UBP2L_HUMAN | 608 | SSISSSPQK | S |
| sp\|Q14203\|DCTN1_HUMAN | 104 | GADTTSPET | T |
| sp\|Q14241\|ELOA1_HUMAN | 542 | KAFSSPQEE | S |
| sp\|Q14247\|SRC8_HUMAN | 405 | TPPVSPAPQ | S |
| sp\|Q14247\|SRC8_HUMAN | 432 | KAELSYRGP | S |
| sp\|Q14247\|SRC8_HUMAN | 401 | AKTQTPPVS | T |
| sp\|Q14432\|PDE3A_HUMAN | 312 | HRRTSLPCI | S |
| sp\|Q14432\|PDE3A_HUMAN | 402 | VTSLSENYT | S |
| sp\|Q14432\|PDE3A_HUMAN | 520 | LAKISPLSS | S |
| sp\|Q14432\|PDE3A_HUMAN | 408 | NYTCSDSEE | S |
| sp\|Q14432\|PDE3A_HUMAN | 406 | SENYTCSDS | T |
| sp\|Q14432\|PDE3A_HUMAN | 311 | SHRRTSLPC | T |
| sp\|Q14498\|RBM39_HUMAN | 136 | RKDKSPVRE | S |
| sp\|Q14573\|ITPR3_HUMAN | 1832 | GRVASFSIP | S |
| sp\|Q14643\|ITPR1_HUMAN | 1603 | VLAASRDYR | S |
| sp\|Q14676\|MDC1_HUMAN | 1400 | KNRSSGKTP | S |
| sp\|Q14676\|MDC1_HUMAN | 1399 | RKNRSSGKT | S |
| sp\|Q14678\|KANK1_HUMAN | 186 | PRLASFGGM | S |
| sp\|Q14684\|RRP1B_HUMAN | 392 | EEEDSESSL | S |
| sp\|Q14687\|GSE1_HUMAN | 907 | ADSLTNSPR | T |
| sp\|Q14847\|LASP1_HUMAN | 104 | VVADTPELQ | T |
| sp\|Q14938\|NFIX_HUMAN | 268 | RSITSPPST | S |
| sp\|Q14938\|NFIX_HUMAN | 265 | LGRRSITSP | S |
| sp\|Q14938\|NFIX_HUMAN | 267 | RRSITSPPS | T |
| sp\|Q14980\|NUMA1_HUMAN | 1834 | SANSSFYST | S |
| sp\|Q14980\|NUMA1_HUMAN | 2074 | KKALSKASP | S |
| sp\|Q14980\|NUMA1_HUMAN | 2077 | LSKASPNTR | S |
| sp\|Q14980\|NUMA1_HUMAN | 1833 | DSANSSFYS | S |
| sp\|Q14C86\|GAPD1_HUMAN | 66 | SAEASPAEC | S |
| sp\|Q15003\|CND2_HUMAN | 201 | VADGSATEM | S |
| sp\|Q15021\|CND1_HUMAN | 1333 | ASTASDNDF | S |
| sp\|Q15021\|CND1_HUMAN | 1339 | NDFVTPEPR | T |
| sp\|Q15021\|CND1_HUMAN | 1331 | PLASTASDN | T |
| sp\|Q15021\|CND1_HUMAN | 1315 | QRAPSAKKP | S |
| sp\|Q15021\|CND1_HUMAN | 1330 | QPLASTASD | S |
| sp\|Q15021\|CND1_HUMAN | 1310 | GQAGSQRAP | S |
| sp\|Q15025\|TNIP1_HUMAN | 403 | QDQLSPLTR | S |
| sp\|Q15054\|DPOD3_HUMAN | 307 | RVALSDDET | S |
| sp\|Q15059\|BRD3_HUMAN | 259 | AITASRSES | S |
| sp\|Q15111\|PLCL1_HUMAN | 77 | PRRSSIIKD | S |
| sp\|Q15149\|PLEC_HUMAN | 4384 | FRSRSSSVG | S |
| sp\|Q15149\|PLEC_HUMAN | 4668 | YGRRYASGS | Y |
| sp\|Q15154\|PCM1_HUMAN | 384 | SRKPSASER | S |
| sp\|Q15154\|PCM1_HUMAN | 68 | ISPESSPGV | S |
| sp\|Q15154\|PCM1_HUMAN | 61 | DKRVTNDIS | T |
| sp\|Q15311\|RBP1_HUMAN | 29 | TRTPSSEEI | S |
| sp\|Q15424\|SAFB1_HUMAN | 604 | RSVVSFDKV | S |
| sp\|Q15424\|SAFB1_HUMAN | 601 | REKRSVVSF | S |
| sp\|Q15459\|SF3A1_HUMAN | 451 | REKQSDDEV | S |
| sp\|Q15637\|SF01_HUMAN | 82 | DRSPSPEPI | S |
| sp\|Q15642\|CIP4_HUMAN | 296 | NRAPSDSSL | S |
| sp\|Q15652\|JHD2C_HUMAN | 639 | KIKSSPSPE | S |
| sp\|Q15652\|JHD2C_HUMAN | 638 | HKIKSSPSP | S |
| sp\|Q15746\|MYLK_HUMAN | 1760 | GRLSSMAMI | S |
| sp\|Q15746\|MYLK_HUMAN | 1759 | IGRLSSMAM | S |
| sp\|Q15751\|HERC1_HUMAN | 2720 | GRRQSLTSP | S |
| sp\|Q15910\|EZH2_HUMAN | 487 | EDVDTPPRK | T |
| sp\|Q15942\|ZYX_HUMAN | 259 | PPASSPAPA | S |
| sp\|Q15942\|ZYX_HUMAN | 267 | APKFSPVTP | S |
| sp\|Q15942\|ZYX_HUMAN | 270 | FSPVTPKFT | T |
| sp\|Q16637\|SMN_HUMAN | 63 | ICETSGKPK | S |
| sp\|Q16643\|DREB_HUMAN | 274 | KKSESEVEE | S |
| sp\|Q16799\|RTN1_HUMAN | 352 | KGSISEDEL | S |
| sp\|Q27J81\|INF2_HUMAN | 1179 | DEEDTAPES | T |
| sp\|Q2PPJ7\|RGPA2_HUMAN | 486 | GRTYSFTSA | S |
| sp\|Q2PPJ7\|RGPA2_HUMAN | 484 | SWGRTYSFT | T |
| sp\|Q3KQU3\|MA7D1_HUMAN | 442 | KKRQSLPAS | S |
| sp\|Q3KQU3\|MA7D1_HUMAN | 454 | RLSASTASE | S |
| sp\|Q3KQU3\|MA7D1_HUMAN | 517 | EAKESPSAA | S |
| sp\|Q3KQU3\|MA7D1_HUMAN | 460 | ASELSPKSK | S |
| sp\|Q3KQU3\|MA7D1_HUMAN | 316 | RSAVTLPRN | T |
| sp\|Q3KQU3\|MA7D1_HUMAN | 457 | ASTASELSP | S |
| sp\|Q3TC46\|PATL1_MOUSE | 179 | RRSTSPIIG | S |
| sp\|Q3TC46\|PATL1_MOUSE | 177 | LPRRSTSPI | S |
| sp\|Q3TLH4\|PRC2C_MOUSE | 2625 | IKPGTPPIG | T |
| sp\|Q49A26\|GLYR1_HUMAN | 130 | KRKLSLSEG | S |
| sp\|Q4G0J3\|LARP7_HUMAN | 337 | DIEISTEEE | S |
| sp\|Q4G0J3\|LARP7_HUMAN | 338 | IEISTEEEK | T |
| sp\|Q4G0J3\|LARP7_HUMAN | 261 | TSEGSDIES | S |
| sp\|Q4KMP7\|TB10B_HUMAN | 658 | GPSSSLLSL | S |
| sp\|Q52LW3\|RHG29_HUMAN | 1144 | ERRSSDSYP | S |
| sp\|Q53EU6\|GPAT3_HUMAN | 68 | KNSASVGII | S |
| sp\|Q53GL0\|PKHO1_HUMAN | 271 | GRCASLEEI | S |
| sp\|Q53HL2\|BOREA_HUMAN | 189 | TPGLTPRFD | T |
| sp\|Q53HL2\|BOREA_HUMAN | 185 | MVKPTPGLT | T |
| sp\|Q53LP3\|SWAHC_HUMAN | 213 | VMGSSPQLK | S |
| sp\|Q58WW2\|DCAF6_HUMAN | 336 | DGEQSPNVS | S |
| sp\|Q58WW2\|DCAF6_HUMAN | 292 | RELKTPSAE | T |
| sp\|Q5H9R7\|PP6R3_HUMAN | 617 | DDGGSDEED | S |
| sp\|Q5JSH3\|WDR44_HUMAN | 199 | SDSLSTKDF | S |
| sp\|Q5JSH3\|WDR44_HUMAN | 96 | QATASPIVA | S |
| sp\|Q5JSH3\|WDR44_HUMAN | 50 | VGNESPVQE | S |
| sp\|Q5JSZ5\|PRC2B_HUMAN | 1808 | GPTGSPVVK | S |
| sp\|Q5JTD0\|TJAP1_HUMAN | 320 | YPTPSPPHP | S |
| sp\|Q5JTD0\|TJAP1_HUMAN | 318 | NPYPTPSPP | T |
| sp\|Q5JTD0\|TJAP1_HUMAN | 316 | KLNPYPTPS | Y |
| sp\|Q5QJE6\|TDIF2_HUMAN | 117 | LIACSPVSS | S |
| sp\|Q5SW79\|CE170_HUMAN | 359 | KSIKSDVPV | S |
| sp\|Q5SW79\|CE170_HUMAN | 920 | EGPDTPSYN | T |
| sp\|Q5SW79\|CE170_HUMAN | 644 | RRRRTLPQL | T |
| sp\|Q5SYE7\|NHSL1_HUMAN | 1089 | VRKNSGAEA | S |
| sp\|Q5T035\|CI129_HUMAN | 66 | TEQNSYSNI | S |
| sp\|Q5T0F9\|C2D1B_HUMAN | 209 | SQLASVRRG | S |
| sp\|Q5T1M5\|FKB15_HUMAN | 356 | AINTSPDAV | S |
| sp\|Q5T1M5\|FKB15_HUMAN | 346 | TKSNSLSEQ | S |
| sp\|Q5T2D3\|OTUD3_HUMAN | 224 | KGMDSEDDL | S |
| sp\|Q5T4S7\|UBR4_HUMAN | 2719 | TLPSSPRSN | S |
| sp\|Q5T4S7\|UBR4_HUMAN | 2718 | VTLPSSPRS | S |
| sp\|Q5T4S7\|UBR4_HUMAN | 2715 | RRHVTLPSS | T |
| sp\|Q5T5C0\|STXB5_HUMAN | 759 | SRKLSLPTD | S |
| sp\|Q5T5P2\|SKT_HUMAN | 1794 | FKPTSPSLP | S |
| sp\|Q5T5P2\|SKT_HUMAN | 1896 | SFSSSPPSP | S |
| sp\|Q5T5P2\|SKT_HUMAN | 1899 | SSPPSPASS | S |
| sp\|Q5T5P2\|SKT_HUMAN | 552 | YSTATIPKD | T |
| sp\|Q5T5P2\|SKT_HUMAN | 1793 | DFKPTSPSL | T |
| sp\|Q5T6F2\|UBAP2_HUMAN | 469 | LRESTPGDS | T |
| sp\|Q5THJ4\|VP13D_HUMAN | 1761 | DYPLTPPPS | T |
| sp\|Q5THJ4\|VP13D_HUMAN | 1765 | TPPPSPTVD | S |
| sp\|Q5U651\|RAIN_HUMAN | 50 | KSSSSDTGS | S |
| sp\|Q5U651\|RAIN_HUMAN | 44 | PSAASVKSS | S |
| sp\|Q5U651\|RAIN_HUMAN | 47 | ASVKSSSSD | S |
| sp\|Q5U651\|RAIN_HUMAN | 331 | VSELSLQGR | S |
| sp\|Q5UIP0\|RIF1_HUMAN | 1693 | FDNCSLGES | S |
| sp\|Q5VT52\|RPRD2_HUMAN | 1099 | NRRMSGEPI | S |
| sp\|Q5VY43\|PEAR1_HUMAN | 953 | PPSGSPPRQ | S |
| sp\|Q5VYK3\|ECM29_HUMAN | 830 | LPIPSEGSG | S |
| sp\|Q5VZ18\|SHE_HUMAN | 69 | LRKNSEAGG | S |
| sp\|Q5VZK9\|CARL1_HUMAN | 916 | TCMMTPKSK | T |
| sp\|Q5VZL5\|ZMYM4_HUMAN | 122 | TQHESDNEN | S |
| sp\|Q60902\|EP15R_MOUSE | 255 | TGSLSPKHS | S |
| sp\|Q62136\|PTN21_MOUSE | 590 | YISSSNPDL | S |
| sp\|Q62188\|DPYL3_MOUSE | 514 | PKGGTPAGS | T |
| sp\|Q63918\|CAVN2_MOUSE | 287 | SSGKSSPFK | S |
| sp\|Q63918\|CAVN2_MOUSE | 288 | SGKSSPFKV | S |
| sp\|Q63ZY3\|KANK2_HUMAN | 540 | ERVPSVAEA | S |
| sp\|Q63ZY3\|KANK2_HUMAN | 375 | GRPESPPVF | S |
| sp\|Q66K74\|MAP1S_HUMAN | 770 | SSARSQERA | S |
| sp\|Q66K74\|MAP1S_HUMAN | 759 | PAPASPGSS | S |
| sp\|Q66K74\|MAP1S_HUMAN | 657 | RLSLSPLRG | S |
| sp\|Q6KC79\|NIPBL_HUMAN | 150 | TISHSPSSR | S |
| sp\|Q6KC79\|NIPBL_HUMAN | 136 | SMHSSPASS | S |
| sp\|Q6KC79\|NIPBL_HUMAN | 135 | NSMHSSPAS | S |
| sp\|Q6P0Q8\|MAST2_HUMAN | 148 | SLGQSAPSL | S |
| sp\|Q6P1Q9\|MET2B_HUMAN | 154 | HKTQTPPVE | T |
| sp\|Q6P1Q9\|MET2B_HUMAN | 152 | LEHKTQTPP | T |
| sp\|Q6P2E9\|EDC4_HUMAN | 879 | SAEQSDHDD | S |
| sp\|Q6P582\|MZT2A_HUMAN | 139 | PRQPSATRL | S |
| sp\|Q6PJW8\|CNST_HUMAN | 68 | SEQDSLNNN | S |
| sp\|Q6PJW8\|CNST_HUMAN | 436 | EPLISPGCD | S |
| sp\|Q6PKG0\|LARP1_HUMAN | 526 | PRAVTPVPT | T |
| sp\|Q6R327\|RICTR_HUMAN | 1320 | DCNFSYTSS | S |
| sp\|Q6UN15\|FIP1_HUMAN | 492 | ERDHSPTPS | S |
| sp\|Q6UN15\|FIP1_HUMAN | 496 | SPTPSVFNS | S |
| sp\|Q6UN15\|FIP1_HUMAN | 500 | SVFNSDEER | S |
| sp\|Q6UN15\|FIP1_HUMAN | 494 | DHSPTPSVF | T |
| sp\|Q6VMQ6\|MCAF1_HUMAN | 677 | SPGKTVNDV | T |
| sp\|Q6VY07\|PACS1_HUMAN | 504 | EGVHTPRQK | T |
| sp\|Q6WCQ1\|MPRIP_HUMAN | 619 | EVDRSPGLP | S |
| sp\|Q6ZRS2\|SRCAP_HUMAN | 1172 | RLILSPDMQ | S |
| sp\|Q6ZTN6\|AN13D_HUMAN | 465 | RGPGSPPRT | S |
| sp\|Q6ZTN6\|AN13D_HUMAN | 469 | SPPRTPPAP | T |
| sp\|Q6ZW31\|SYDE1_HUMAN | 222 | GPGGTRSPR | T |
| sp\|Q6ZW31\|SYDE1_HUMAN | 224 | GGTRSPRAG | S |
| sp\|Q71F56\|MD13L_HUMAN | 2103 | YFVSTAKAE | T |
| sp\|Q71F56\|MD13L_HUMAN | 2102 | GYFVSTAKA | S |
| sp\|Q76N33\|STALP_MOUSE | 242 | FANYSPPVN | S |
| sp\|Q7KZI7\|MARK2_HUMAN | 456 | KVPASPLPG | S |
| sp\|Q7L1W4\|LRC8D_HUMAN | 246 | SDEGSPSAS | S |
| sp\|Q7L2J0\|MEPCE_HUMAN | 69 | VGRESPGAA | S |
| sp\|Q7L4E1\|MIGA2_HUMAN | 276 | LTEGSLRLR | S |
| sp\|Q7L4I2\|RSRC2_HUMAN | 30 | QSEVSVSPR | S |
| sp\|Q7L4I2\|RSRC2_HUMAN | 104 | ERLNSSENG | S |
| sp\|Q7L4I2\|RSRC2_HUMAN | 32 | EVSVSPRAS | S |
| sp\|Q7LDG7\|GRP2_HUMAN | 578 | AFSFSLPRP | S |
| sp\|Q7LDG7\|GRP2_HUMAN | 587 | GRRGSRPPE | S |
| sp\|Q7Z333\|SETX_HUMAN | 1330 | TKLISPQNL | S |
| sp\|Q7Z417\|NUFP2_HUMAN | 212 | DNDGSGSES | S |
| sp\|Q7Z417\|NUFP2_HUMAN | 219 | ESGYTTPKK | T |
| sp\|Q7Z434\|MAVS_HUMAN | 222 | RGPVSPSVS | S |
| sp\|Q7Z4S6\|KI21A_HUMAN | 1229 | SRQSSLSEK | S |
| sp\|Q7Z4S6\|KI21A_HUMAN | 1225 | IGSISRQSS | S |
| sp\|Q7Z4V5\|HDGR2_HUMAN | 234 | SASDSDSKA | S |
| sp\|Q7Z4V5\|HDGR2_HUMAN | 232 | APSASDSDS | S |
| sp\|Q7Z4V5\|HDGR2_HUMAN | 240 | SKADSDGAK | S |
| sp\|Q7Z5K2\|WAPL_HUMAN | 223 | PESPSEISP | S |
| sp\|Q7Z5L9\|I2BP2_HUMAN | 457 | RNSNSPPSP | S |
| sp\|Q7Z5L9\|I2BP2_HUMAN | 460 | NSPPSPSSM | S |
| sp\|Q7Z5L9\|I2BP2_HUMAN | 455 | TRRNSNSPP | S |
| sp\|Q7Z6Z7\|HUWE1_HUMAN | 2887 | PRAGSSTPG | S |
| sp\|Q80TV8\|CLAP1_MOUSE | 1193 | FSFRSQEDL | S |
| sp\|Q86SQ0\|PHLB2_HUMAN | 212 | ARKMSIQDS | S |
| sp\|Q86SQ0\|PHLB2_HUMAN | 468 | DSRLSTGTT | S |
| sp\|Q86TB9\|PATL1_HUMAN | 178 | PRRSTSPII | T |
| sp\|Q86TC9\|MYPN_HUMAN | 928 | PVDESDDEI | S |
| sp\|Q86UE4\|LYRIC_HUMAN | 339 | DWGRSWSDR | S |
| sp\|Q86UP2\|KTN1_HUMAN | 204 | TKQESGSGK | S |
| sp\|Q86UU1\|PHLB1_HUMAN | 461 | LPPLSPSLS | S |
| sp\|Q86UU1\|PHLB1_HUMAN | 583 | ITEISDNED | S |
| sp\|Q86UU1\|PHLB1_HUMAN | 430 | GRTFSDGLA | S |
| sp\|Q86UU1\|PHLB1_HUMAN | 578 | ERKNSITEI | S |
| sp\|Q86V48\|LUZP1_HUMAN | 638 | VMEDSSPHE | S |
| sp\|Q86V48\|LUZP1_HUMAN | 957 | TQRSSTDFS | S |
| sp\|Q86V48\|LUZP1_HUMAN | 932 | RDLKSLEDP | S |
| sp\|Q86VM9\|ZCH18_HUMAN | 173 | AGVQSVGEK | S |
| sp\|Q86VM9\|ZCH18_HUMAN | 605 | SFSSSPSPS | S |
| sp\|Q86WB0\|NIPA_HUMAN | 335 | DATFSPGSE | S |
| sp\|Q86WB0\|NIPA_HUMAN | 344 | QAEKSPGPI | S |
| sp\|Q86WB0\|NIPA_HUMAN | 333 | SQDATFSPG | T |
| sp\|Q86WR7\|PRSR2_HUMAN | 312 | GGGSSPERV | S |
| sp\|Q86YP4\|P66A_HUMAN | 107 | VIVLSDNEQ | S |
| sp\|Q86YP4\|P66A_HUMAN | 100 | RRPPSPDVI | S |
| sp\|Q8BGU5\|CCNY_MOUSE | 73 | FLSKSQTDV | S |
| sp\|Q8C052\|MAP1S_MOUSE | 658 | LARRSTSPH | S |
| sp\|Q8CFH6\|SIK2_MOUSE | 587 | GRRASDTSL | S |
| sp\|Q8IUW5\|RELL1_HUMAN | 244 | KERRSLMSV | S |
| sp\|Q8IWW6\|RHG12_HUMAN | 215 | QDSESGDEL | S |
| sp\|Q8IWZ3\|ANKH1_HUMAN | 1591 | AGGNSDSDN | S |
| sp\|Q8IWZ3\|ANKH1_HUMAN | 1593 | GNSDSDNLD | S |
| sp\|Q8IX01\|SUGP2_HUMAN | 705 | QTLLSSGTR | S |
| sp\|Q8IYA6\|CKP2L_HUMAN | 320 | TKIRSYPVT | S |
| sp\|Q8IYB3\|SRRM1_HUMAN | 695 | PQTSSSPPP | S |
| sp\|Q8IYB3\|SRRM1_HUMAN | 391 | RLSPSASPP | S |
| sp\|Q8IYB3\|SRRM1_HUMAN | 393 | SPSASPPRR | S |
| sp\|Q8IYB3\|SRRM1_HUMAN | 607 | RYSPSPPPK | S |
| sp\|Q8IYB3\|SRRM1_HUMAN | 389 | TRRLSPSAS | S |
| sp\|Q8IYB3\|SRRM1_HUMAN | 463 | KVELSESEE | S |
| sp\|Q8IYB3\|SRRM1_HUMAN | 465 | ELSESEEDK | S |
| sp\|Q8IYB3\|SRRM1_HUMAN | 754 | SRSVSGSPE | S |
| sp\|Q8IYB3\|SRRM1_HUMAN | 694 | APQTSSSPP | S |
| sp\|Q8IYB3\|SRRM1_HUMAN | 756 | SVSGSPEPA | S |
| sp\|Q8IYB3\|SRRM1_HUMAN | 872 | PKKETESEA | T |
| sp\|Q8IYB3\|SRRM1_HUMAN | 604 | IQRRYSPSP | Y |
| sp\|Q8IYB3\|SRRM1_HUMAN | 874 | KETESEAED | S |
| sp\|Q8IZ21\|PHAR4_HUMAN | 427 | QALTSPLPM | S |
| sp\|Q8IZ21\|PHAR4_HUMAN | 432 | PLPMTPILE | T |
| sp\|Q8IZD0\|SAM14_HUMAN | 117 | EPPPSPLTR | S |
| sp\|Q8IZP0\|ABI1_HUMAN | 225 | GSQHSPGRT | S |
| sp\|Q8IZT6\|ASPM_HUMAN | 283 | TSFNSVNVN | S |
| sp\|Q8IZT6\|ASPM_HUMAN | 280 | VTETSFNSV | S |
| sp\|Q8IZT6\|ASPM_HUMAN | 170 | NRRVSNIQN | S |
| sp\|Q8IZT6\|ASPM_HUMAN | 35 | EEASSPPVL | S |
| sp\|Q8K124\|PKHO2_MOUSE | 395 | PRSSSLGDL | S |
| sp\|Q8N392\|RHG18_HUMAN | 66 | DRSISQDSL | S |
| sp\|Q8N392\|RHG18_HUMAN | 260 | GDDATLPSF | T |
| sp\|Q8N3D4\|EH1L1_HUMAN | 1168 | SAQPSPPDD | S |
| sp\|Q8N3D4\|EH1L1_HUMAN | 285 | PPETSPEMR | S |
| sp\|Q8N556\|AFAP1_HUMAN | 679 | TLENSPISS | S |
| sp\|Q8N5A5\|ZGPAT_HUMAN | 373 | PRGKSLDQC | S |
| sp\|Q8N699\|MYCT1_HUMAN | 107 | ISQWSSSRR | S |
| sp\|Q8N6N3\|CA052_HUMAN | 158 | ETLESDDEK | S |
| sp\|Q8N6N3\|CA052_HUMAN | 155 | EGEETLESD | T |
| sp\|Q8N9U0\|TAC2N_HUMAN | 174 | GLSKSMFDL | S |
| sp\|Q8NC56\|LEMD2_HUMAN | 453 | IPPQSRRRM | S |
| sp\|Q8NCF5\|NF2IP_HUMAN | 90 | DNSNSDSEG | S |
| sp\|Q8NCF5\|NF2IP_HUMAN | 92 | SNSDSEGED | S |
| sp\|Q8ND82\|Z280C_HUMAN | 227 | TNTSSPYDA | S |
| sp\|Q8NDI1\|EHBP1_HUMAN | 174 | QSLASLMSM | S |
| sp\|Q8NDI1\|EHBP1_HUMAN | 171 | EDMQSLASL | S |
| sp\|Q8NE71\|ABCF1_HUMAN | 109 | SVPTSDEED | S |
| sp\|Q8NE71\|ABCF1_HUMAN | 108 | LSVPTSDEE | T |
| sp\|Q8NEC7\|GSTCD_HUMAN | 232 | EGLDSSSKS | S |
| sp\|Q8NEJ9\|NGDN_HUMAN | 214 | KEQYSDAPE | S |
| sp\|Q8NEJ9\|NGDN_HUMAN | 213 | LKEQYSDAP | Y |
| sp\|Q8NEY1\|NAV1_HUMAN | 648 | GRKTSLDVS | S |
| sp\|Q8NEY1\|NAV1_HUMAN | 199 | EDGKSDDEL | S |
| sp\|Q8NEY1\|NAV1_HUMAN | 1381 | SSALSSPRR | S |
| sp\|Q8NF91\|SYNE1_HUMAN | 8305 | RALPSEDEE | S |
| sp\|Q8NFC6\|BD1L1_HUMAN | 1316 | GSTASTSPA | S |
| sp\|Q8NFC6\|BD1L1_HUMAN | 2964 | DAESSEPER | S |
| sp\|Q8NFC6\|BD1L1_HUMAN | 660 | RRTSTPVIM | T |
| sp\|Q8NFC6\|BD1L1_HUMAN | 2956 | KRSLTVSDD | T |
| sp\|Q8NFQ8\|TOIP2_HUMAN | 188 | PEAGSHPQQ | S |
| sp\|Q8NFQ8\|TOIP2_HUMAN | 120 | DPSHSPSDK | S |
| sp\|Q8TB72\|PUM2_HUMAN | 587 | TRRESLSTS | S |
| sp\|Q8TC07\|TBC15_HUMAN | 675 | ARNDSPTQI | S |
| sp\|Q8TD16\|BICD2_HUMAN | 379 | HTRGSLSEQ | S |
| sp\|Q8TD55\|PKHO2_HUMAN | 390 | PRCSSLGDL | S |
| sp\|Q8TD55\|PKHO2_HUMAN | 224 | DRVETPVGE | T |
| sp\|Q8TDJ6\|DMXL2_HUMAN | 1400 | SRTISVSGS | S |
| sp\|Q8TED9\|AF1L1_HUMAN | 747 | KRSPSIVAS | S |
| sp\|Q8TEH3\|DEN1A_HUMAN | 707 | QGRKTPELG | T |
| sp\|Q8TF01\|PNISR_HUMAN | 290 | SKFDSDEEE | S |
| sp\|Q8WUM0\|NU133_HUMAN | 31 | STPRTASRK | T |
| sp\|Q8WUM0\|NU133_HUMAN | 28 | GPGSTPRTA | T |
| sp\|Q8WVV9\|HNRLL_HUMAN | 35 | EIDYSAEEG | S |
| sp\|Q8WVV9\|HNRLL_HUMAN | 34 | GEIDYSAEE | Y |
| sp\|Q8WW12\|PCNP_HUMAN | 119 | EDEDSEPEE | S |
| sp\|Q8WWI1\|LMO7_HUMAN | 246 | KREDSFESL | S |
| sp\|Q8WWI1\|LMO7_HUMAN | 751 | DRRKSYTSD | S |
| sp\|Q8WWI1\|LMO7_HUMAN | 1493 | QRSASVNKE | S |
| sp\|Q8WWI1\|LMO7_HUMAN | 276 | EGFESDTDS | S |
| sp\|Q8WWI1\|LMO7_HUMAN | 278 | FESDTDSEF | T |
| sp\|Q8WWM7\|ATX2L_HUMAN | 680 | NKSTSTPTS | S |
| sp\|Q8WWM7\|ATX2L_HUMAN | 684 | STPTSPGPR | S |
| sp\|Q8WWM7\|ATX2L_HUMAN | 339 | SGRESPSLA | S |
| sp\|Q8WWQ0\|PHIP_HUMAN | 676 | RGSISSTSE | S |
| sp\|Q8WWQ0\|PHIP_HUMAN | 674 | LSRGSISST | S |
| sp\|Q8WXG6\|MADD_HUMAN | 1270 | KLAGSPIRT | S |
| sp\|Q8WXG6\|MADD_HUMAN | 1245 | SEIETNSAT | T |
| sp\|Q8WXG6\|MADD_HUMAN | 1247 | IETNSATST | S |
| sp\|Q8WXI9\|P66B_HUMAN | 135 | NEASSPRSS | S |
| sp\|Q8WXI9\|P66B_HUMAN | 122 | RLTPSPDII | S |
| sp\|Q8WXI9\|P66B_HUMAN | 134 | DNEASSPRS | S |
| sp\|Q8WXI9\|P66B_HUMAN | 129 | IIVLSDNEA | S |
| sp\|Q8WXI9\|P66B_HUMAN | 120 | RGRLTPSPD | T |
| sp\|Q921I6\|SH3B4_MOUSE | 278 | EQFQSREDF | S |
| sp\|Q92508\|PIEZ1_HUMAN | 1395 | SPGGSSPPR | S |
| sp\|Q92508\|PIEZ1_HUMAN | 1396 | PGGSSPPRR | S |
| sp\|Q92508\|PIEZ1_HUMAN | 167 | DASPTAGLQ | T |
| sp\|Q92536\|YLAT2_HUMAN | 19 | LVPNTSQSQ | T |
| sp\|Q92538\|GBF1_HUMAN | 1298 | AGAQSDSEL | S |
| sp\|Q92574\|TSC1_HUMAN | 505 | GGFDSPFYR | S |
| sp\|Q92597\|NDRG1_HUMAN | 336 | SSVTSLDGT | S |
| sp\|Q92597\|NDRG1_HUMAN | 333 | ASGSSVTSL | S |
| sp\|Q92597\|NDRG1_HUMAN | 330 | SRTASGSSV | S |
| sp\|Q92597\|NDRG1_HUMAN | 335 | GSSVTSLDG | T |
| sp\|Q92597\|NDRG1_HUMAN | 328 | MRSRTASGS | T |
| sp\|Q92609\|TBCD5_HUMAN | 42 | NGRRTSSTL | T |
| sp\|Q92615\|LAR4B_HUMAN | 451 | NGVRSPQTR | S |
| sp\|Q92619\|HMHA1_HUMAN | 569 | ANAWSPVMR | S |
| sp\|Q92619\|HMHA1_HUMAN | 25 | AGSPSPQPS | S |
| sp\|Q92619\|HMHA1_HUMAN | 23 | NRAGSPSPQ | S |
| sp\|Q92620\|PRP16_HUMAN | 513 | MKRKSEASS | S |
| sp\|Q92625\|ANS1A_HUMAN | 626 | SLSKSDSDL | S |
| sp\|Q92733\|PRCC_HUMAN | 159 | GDSDSEEDE | S |
| sp\|Q92854\|SEM4D_HUMAN | 840 | IDDLSARDK | S |
| sp\|Q92945\|FUBP2_HUMAN | 181 | KVQISPDSG | S |
| sp\|Q92945\|FUBP2_HUMAN | 184 | ISPDSGGLP | S |
| sp\|Q93100\|KPBB_HUMAN | 27 | KRSGSVYEP | S |
| sp\|Q96A00\|PP14A_HUMAN | 26 | GPGGSPGGL | S |
| sp\|Q96A57\|TM230_HUMAN | 24 | SRLSSTDDG | S |
| sp\|Q96AE4\|FUBP1_HUMAN | 268 | VRNEYGSRI | Y |
| sp\|Q96AP7\|ESAM_HUMAN | 359 | PQPISPIPG | S |
| sp\|Q96AT1\|K1143_HUMAN | 50 | DGDHSDKED | S |
| sp\|Q96B36\|AKTS1_HUMAN | 203 | EARSSDEEN | S |
| sp\|Q96B36\|AKTS1_HUMAN | 183 | QYAKSLPVS | S |
| sp\|Q96B36\|AKTS1_HUMAN | 202 | TEARSSDEE | S |
| sp\|Q96C24\|SYTL4_HUMAN | 274 | EYTKSVIDL | S |
| sp\|Q96C24\|SYTL4_HUMAN | 296 | DRSKSVPGL | S |
| sp\|Q96C24\|SYTL4_HUMAN | 217 | SRRDSLDKS | S |
| sp\|Q96H79\|ZCCHL_HUMAN | 257 | TDNSSPSTE | S |
| sp\|Q96H79\|ZCCHL_HUMAN | 260 | SSPSTEHSQ | T |
| sp\|Q96HN2\|SAHH3_HUMAN | 107 | EALVSPDGT | S |
| sp\|Q96JM3\|CHAP1_HUMAN | 436 | SPAGSPELR | S |
| sp\|Q96JM3\|CHAP1_HUMAN | 432 | PEIRSPAGS | S |
| sp\|Q96K49\|TM87B_HUMAN | 496 | SKSVSNGTA | S |
| sp\|Q96L73\|NSD1_HUMAN | 483 | LAFDSEHSA | S |
| sp\|Q96L73\|NSD1_HUMAN | 486 | DSEHSADEK | S |
| sp\|Q96MK2\|RIPR3_HUMAN | 832 | QLDGTPRVC | T |
| sp\|Q96MK2\|RIPR3_HUMAN | 384 | LSYLSDSDL | S |
| sp\|Q96NT1\|NP1L5_HUMAN | 76 | SLPNSVKCR | S |
| sp\|Q96P48\|ARAP1_HUMAN | 229 | EFDDSDYDE | S |
| sp\|Q96P48\|ARAP1_HUMAN | 231 | DDSDYDEVP | Y |
| sp\|Q96PK6\|RBM14_HUMAN | 620 | RLSESQLSF | S |
| sp\|Q96PN7\|TREF1_HUMAN | 619 | KPASSMSDD | S |
| sp\|Q96PN7\|TREF1_HUMAN | 621 | ASSMSDDEM | S |
| sp\|Q96R06\|SPAG5_HUMAN | 397 | PQEKSTNTS | S |
| sp\|Q96RU3\|FNBP1_HUMAN | 301 | DNSLSNSRG | S |
| sp\|Q96RU3\|FNBP1_HUMAN | 296 | KRTVSDNSL | S |
| sp\|Q96ST2\|IWS1_HUMAN | 415 | SRVVSDADD | S |
| sp\|Q96ST2\|IWS1_HUMAN | 398 | AAVLSDSED | S |
| sp\|Q96ST2\|IWS1_HUMAN | 235 | RHQASDSEN | S |
| sp\|Q96ST2\|IWS1_HUMAN | 420 | DADDSDSDA | S |
| sp\|Q96ST2\|IWS1_HUMAN | 237 | QASDSENEE | S |
| sp\|Q96ST2\|IWS1_HUMAN | 400 | VLSDSEDEE | S |
| sp\|Q96T23\|RSF1_HUMAN | 516 | DADSSISVL | S |
| sp\|Q99426\|TBCB_HUMAN | 110 | KYTISQEAY | S |
| sp\|Q99501\|GA2L1_HUMAN | 306 | PRPASPVPG | S |
| sp\|Q99501\|GA2L1_HUMAN | 489 | LSRVSSPSP | S |
| sp\|Q99570\|PI3R4_HUMAN | 813 | HLHDSSQKG | S |
| sp\|Q99618\|CDCA3_HUMAN | 199 | VLGRSPLTI | S |
| sp\|Q99618\|CDCA3_HUMAN | 209 | QDDNSPGTL | S |
| sp\|Q99618\|CDCA3_HUMAN | 202 | RSPLTILQD | T |
| sp\|Q99627\|CSN8_HUMAN | 175 | ALDVSFNKF | S |
| sp\|Q99683\|M3K5_HUMAN | 1029 | FEDHSAPPS | S |
| sp\|Q99683\|M3K5_HUMAN | 1033 | SAPPSPEEK | S |
| sp\|Q99733\|NP1L4_HUMAN | 304 | GDGESLDED | S |
| sp\|Q99PL6\|UBXN6_MOUSE | 36 | TKGKSPQLA | S |
| sp\|Q9BPX3\|CND3_HUMAN | 674 | TEINSDDEQ | S |
| sp\|Q9BQ39\|DDX50_HUMAN | 113 | KRVSSLDTS | S |
| sp\|Q9BQ52\|RNZ2_HUMAN | 217 | SDSESNENE | S |
| sp\|Q9BQ52\|RNZ2_HUMAN | 208 | LSRLSPERS | S |
| sp\|Q9BQ52\|RNZ2_HUMAN | 212 | SPERSSDSE | S |
| sp\|Q9BQ52\|RNZ2_HUMAN | 213 | PERSSDSES | S |
| sp\|Q9BQG0\|MBB1A_HUMAN | 1293 | KSPLSALAR | S |
| sp\|Q9BQG0\|MBB1A_HUMAN | 1290 | VLGKSPLSA | S |
| sp\|Q9BTC0\|DIDO1_HUMAN | 154 | DTSDSDSDG | S |
| sp\|Q9BTC0\|DIDO1_HUMAN | 152 | HDDTSDSDS | S |
| sp\|Q9BTU6\|P4K2A_HUMAN | 462 | QRSSSESYT | S |
| sp\|Q9BTU6\|P4K2A_HUMAN | 461 | HQRSSSESY | S |
| sp\|Q9BW04\|SARG_HUMAN | 456 | NSALTPPKP | T |
| sp\|Q9BW71\|HIRP3_HUMAN | 227 | SEQESEEEI | S |
| sp\|Q9BW71\|HIRP3_HUMAN | 291 | GDSDSEEEQ | S |
| sp\|Q9BW71\|HIRP3_HUMAN | 289 | LLGDSDSEE | S |
| sp\|Q9BW71\|HIRP3_HUMAN | 223 | SLKESEQES | S |
| sp\|Q9BX40\|LS14B_HUMAN | 154 | PVGKSPMVE | S |
| sp\|Q9BXB4\|OSB11_HUMAN | 189 | QRRPSQNAI | S |
| sp\|Q9BY89\|K1671_HUMAN | 1224 | QERRSPTVE | S |
| sp\|Q9BYW2\|SETD2_HUMAN | 323 | LGSESDEDS | S |
| sp\|Q9BZL6\|KPCD2_HUMAN | 710 | SFRRSVVGT | S |
| sp\|Q9C0C2\|TB182_HUMAN | 429 | QRRFSEGVL | S |
| sp\|Q9H040\|SPRTN_HUMAN | 268 | SNLPSPGKL | S |
| sp\|Q9H0H5\|RGAP1_HUMAN | 214 | QGNESIVAK | S |
| sp\|Q9H1E3\|NUCKS_HUMAN | 130 | QEKDSGSDE | S |
| sp\|Q9H1E3\|NUCKS_HUMAN | 132 | KDSGSDEDF | S |
| sp\|Q9H1E3\|NUCKS_HUMAN | 79 | SAEDSEDEK | S |
| sp\|Q9H1E3\|NUCKS_HUMAN | 144 | DDDDSDYGS | S |
| sp\|Q9H2G2\|SLK_HUMAN | 779 | KDSGSISLQ | S |
| sp\|Q9H2Y7\|ZN106_HUMAN | 864 | LSESSVIMD | S |
| sp\|Q9H2Y7\|ZN106_HUMAN | 861 | KRSLSESSV | S |
| sp\|Q9H3N1\|TMX1_HUMAN | 247 | EEDVSEEEA | S |
| sp\|Q9H425\|CA198_HUMAN | 289 | GKLPSPDVR | S |
| sp\|Q9H4A3\|WNK1_HUMAN | 1261 | RFIVSPVPE | S |
| sp\|Q9H4G0\|E41L1_HUMAN | 546 | PASPSPKGT | S |
| sp\|Q9H4G0\|E41L1_HUMAN | 550 | SPKGTPEKA | T |
| sp\|Q9H4L4\|SENP3_HUMAN | 54 | PDPGSGTTV | S |
| sp\|Q9H4L4\|SENP3_HUMAN | 44 | PRLKSGGGF | S |
| sp\|Q9H6S0\|YTDC2_HUMAN | 1221 | VLMKSPSPA | S |
| sp\|Q9H6S0\|YTDC2_HUMAN | 1223 | MKSPSPALH | S |
| sp\|Q9H6Z4\|RANB3_HUMAN | 108 | GGEDSDRED | S |
| sp\|Q9H6Z4\|RANB3_HUMAN | 124 | KRERTSSLT | T |
| sp\|Q9H6Z4\|RANB3_HUMAN | 101 | AGGSSPEGG | S |
| sp\|Q9H832\|UBE2Z_HUMAN | 337 | AEMDSDSSS | S |
| sp\|Q9H8S9\|MOB1A_HUMAN | 38 | ATLGSGNLR | S |
| sp\|Q9H8S9\|MOB1A_HUMAN | 35 | HAEATLGSG | T |
| sp\|Q9H910\|JUPI2_HUMAN | 132 | KAARSIPAG | S |
| sp\|Q9HBD1\|RC3H2_HUMAN | 982 | HRKHSSTGD | S |
| sp\|Q9HBL0\|TENS1_HUMAN | 1381 | FRQGSPTPA | S |
| sp\|Q9HBL0\|TENS1_HUMAN | 899 | RRAASDGQY | S |
| sp\|Q9HBL0\|TENS1_HUMAN | 1177 | TVPGSPQAR | S |
| sp\|Q9HBL0\|TENS1_HUMAN | 1126 | QPSPSAQRN | S |
| sp\|Q9HC35\|EMAL4_HUMAN | 146 | RASPSPQPS | S |
| sp\|Q9HCD6\|TANC2_HUMAN | 425 | SAHSSITSG | S |
| sp\|Q9HCD6\|TANC2_HUMAN | 427 | HSSITSGSC | T |
| sp\|Q9HD20\|AT131_HUMAN | 905 | TLSNSGIRA | S |
| sp\|Q9HD20\|AT131_HUMAN | 899 | RPRDSPTLS | S |
| sp\|Q9HD20\|AT131_HUMAN | 946 | EDESTPIVK | T |
| sp\|Q9HD20\|AT131_HUMAN | 945 | LEDESTPIV | S |
| sp\|Q9HD67\|MYO10_HUMAN | 1883 | KRRTSFLEG | S |
| sp\|Q9HD67\|MYO10_HUMAN | 1882 | EKRRTSFLE | T |
| sp\|Q9JLV1\|BAG3_MOUSE | 270 | LRAASPFRS | S |
| sp\|Q9JLV1\|BAG3_MOUSE | 274 | SPFRSPVRG | S |
| sp\|Q9NPI6\|DCP1A_HUMAN | 525 | ASSPSPLTI | S |
| sp\|Q9NQ75\|CASS4_HUMAN | 249 | DTPVSPGKA | S |
| sp\|Q9NQ75\|CASS4_HUMAN | 291 | SRSLTPQLN | T |
| sp\|Q9NQG7\|HPS4_HUMAN | 355 | GLSSSLGKE | S |
| sp\|Q9NQT8\|KI13B_HUMAN | 1797 | TLSGSATNL | S |
| sp\|Q9NQT8\|KI13B_HUMAN | 1644 | PAPGSPFRV | S |
| sp\|Q9NQW6\|ANLN_HUMAN | 90 | QPVESTSAK | S |
| sp\|Q9NQW6\|ANLN_HUMAN | 54 | QQPLSGGEE | S |
| sp\|Q9NRA0\|SPHK2_HUMAN | 404 | ELTLTPDPA | T |
| sp\|Q9NRF8\|PYRG2_HUMAN | 562 | GCKLSSSDR | S |
| sp\|Q9NRF8\|PYRG2_HUMAN | 563 | CKLSSSDRY | S |
| sp\|Q9NRL2\|BAZ1A_HUMAN | 731 | QDMVTEDED | T |
| sp\|Q9NRL3\|STRN4_HUMAN | 206 | LLGRSLELN | S |
| sp\|Q9NSC5\|HOME3_HUMAN | 95 | LGFASEQHL | S |
| sp\|Q9NSC5\|HOME3_HUMAN | 159 | FRSQSADAP | S |
| sp\|Q9NTI5\|PDS5B_HUMAN | 1283 | DEQNSPPKK | S |
| sp\|Q9NTJ3\|SMC4_HUMAN | 27 | PDGASSDAE | S |
| sp\|Q9NTJ3\|SMC4_HUMAN | 28 | DGASSDAEP | S |
| sp\|Q9NTZ6\|RBM12_HUMAN | 526 | QNFSYDQRE | Y |
| sp\|Q9NUQ6\|SPS2L_HUMAN | 338 | AARFSCDIE | S |
| sp\|Q9NUQ6\|SPS2L_HUMAN | 195 | KAKTSPVKS | S |
| sp\|Q9NWH9\|SLTM_HUMAN | 553 | ISSKSPGHM | S |
| sp\|Q9NWH9\|SLTM_HUMAN | 551 | KRISSKSPG | S |
| sp\|Q9NXG2\|THUM1_HUMAN | 88 | QPSGSEGED | S |
| sp\|Q9NXG2\|THUM1_HUMAN | 86 | DQQPSGSEG | S |
| sp\|Q9NXH9\|TRM1_HUMAN | 625 | CYSHSPPTP | S |
| sp\|Q9NXH9\|TRM1_HUMAN | 628 | HSPPTPRVS | T |
| sp\|Q9NXH9\|TRM1_HUMAN | 622 | DQCCYSHSP | Y |
| sp\|Q9NXR1\|NDE1_HUMAN | 309 | PSSTSVPLG | S |
| sp\|Q9NXR1\|NDE1_HUMAN | 306 | ERRPSSTSV | S |
| sp\|Q9NYD6\|HXC10_HUMAN | 189 | EHLESPQLG | S |
| sp\|Q9NYF8\|BCLF1_HUMAN | 397 | KFNDSEGDD | S |
| sp\|Q9NYF8\|BCLF1_HUMAN | 402 | EGDDTEETE | T |
| sp\|Q9NYF8\|BCLF1_HUMAN | 511 | DLFDYSPPL | Y |
| sp\|Q9NYV4\|CDK12_HUMAN | 333 | RRRSSSPFL | S |
| sp\|Q9NYV4\|CDK12_HUMAN | 334 | RRSSSPFLS | S |
| sp\|Q9NYV4\|CDK12_HUMAN | 332 | GRRRSSSPF | S |
| sp\|Q9NYZ3\|GTSE1_HUMAN | 516 | PTPASRRCS | S |
| sp\|Q9NYZ3\|GTSE1_HUMAN | 303 | KAPGSTSNL | S |
| sp\|Q9NYZ3\|GTSE1_HUMAN | 513 | SALPTPASR | T |
| sp\|Q9NZJ0\|DTL_HUMAN | 510 | TRTPSSSPP | S |
| sp\|Q9NZJ0\|DTL_HUMAN | 516 | SPPITPPAS | T |
| sp\|Q9NZM3\|ITSN2_HUMAN | 884 | TRTVSPGSV | S |
| sp\|Q9NZM3\|ITSN2_HUMAN | 889 | PGSVSPIHG | S |
| sp\|Q9NZN8\|CNOT2_HUMAN | 165 | SGLGSPNRS | S |
| sp\|Q9P0N8\|MARH2_HUMAN | 49 | GRLLSTVIR | S |
| sp\|Q9P0V3\|SH3B4_HUMAN | 279 | EQFQSREDF | S |
| sp\|Q9P0V3\|SH3B4_HUMAN | 241 | PFFRSKRSY | S |
| sp\|Q9P266\|JCAD_HUMAN | 1044 | NRGLSAPDL | S |
| sp\|Q9P266\|JCAD_HUMAN | 757 | HRSLSPSSN | S |
| sp\|Q9P270\|SLAI2_HUMAN | 72 | PLGLSAKSG | S |
| sp\|Q9P275\|UBP36_HUMAN | 764 | TLLSSTPKP | S |
| sp\|Q9P275\|UBP36_HUMAN | 546 | PQHFSPRTA | S |
| sp\|Q9P275\|UBP36_HUMAN | 756 | QPPFSPHPT | S |
| sp\|Q9P2K5\|MYEF2_HUMAN | 431 | GFGDSFGRL | S |
| sp\|Q9UBC3\|DNM3B_HUMAN | 136 | HVDESPVEF | S |
| sp\|Q9UBE0\|SAE1_HUMAN | 185 | VAKVSQGVE | S |
| sp\|Q9UBW5\|BIN2_HUMAN | 440 | SPTASGGGS | S |
| sp\|Q9UBW5\|BIN2_HUMAN | 436 | NIPSSPTAS | S |
| sp\|Q9UBW5\|BIN2_HUMAN | 466 | SLEVSPNPE | S |
| sp\|Q9UBW5\|BIN2_HUMAN | 444 | SGGGSPTSP | S |
| sp\|Q9UBW5\|BIN2_HUMAN | 438 | PSSPTASGG | T |
| sp\|Q9UBW5\|BIN2_HUMAN | 425 | RATASPRPS | S |
| sp\|Q9UBW5\|BIN2_HUMAN | 263 | SLVISPPVR | S |
| sp\|Q9UDT6\|CLIP2_HUMAN | 211 | SDSGSVKRG | S |
| sp\|Q9UDT6\|CLIP2_HUMAN | 204 | NESGSNLSD | S |
| sp\|Q9UDT6\|CLIP2_HUMAN | 207 | GSNLSDSGS | S |
| sp\|Q9UDY2\|ZO2_HUMAN | 150 | FDGRSFRSG | S |
| sp\|Q9UDY2\|ZO2_HUMAN | 461 | TPFKSTGDI | S |
| sp\|Q9UEU0\|VTI1B_HUMAN | 103 | PLTATPGGR | T |
| sp\|Q9UEY8\|ADDG_HUMAN | 650 | VSRLSTSTT | S |
| sp\|Q9UGV2\|NDRG3_HUMAN | 331 | SRTHSTSSS | S |
| sp\|Q9UGV2\|NDRG3_HUMAN | 327 | RLARSRTHS | S |
| sp\|Q9UHB6\|LIMA1_HUMAN | 686 | NGADSDEDD | S |
| sp\|Q9UHJ3\|SMBT1_HUMAN | 775 | NKPPSPKEI | S |
| sp\|Q9UHJ3\|SMBT1_HUMAN | 767 | TFSFSDDEN | S |
| sp\|Q9UHR4\|BI2L1_HUMAN | 261 | TPQASPMIE | S |
| sp\|Q9UHR4\|BI2L1_HUMAN | 257 | PVSGTPQAS | T |
| sp\|Q9UHR4\|BI2L1_HUMAN | 255 | STPVSGTPQ | S |
| sp\|Q9UHR5\|S30BP_HUMAN | 43 | GGLVSDAYG | S |
| sp\|Q9UIG0\|BAZ1B_HUMAN | 947 | DHTVSGDED | S |
| sp\|Q9UIG0\|BAZ1B_HUMAN | 189 | GRRESINDR | S |
| sp\|Q9UIG0\|BAZ1B_HUMAN | 945 | CKDHTVSGD | T |
| sp\|Q9UJW0\|DCTN4_HUMAN | 196 | RAGASISTL | S |
| sp\|Q9UJX5\|APC4_HUMAN | 779 | VLSESEAEN | S |
| sp\|Q9UKE5\|TNIK_HUMAN | 680 | TTSISPALA | S |
| sp\|Q9UKE5\|TNIK_HUMAN | 707 | PIRASNPDL | S |
| sp\|Q9UKV3\|ACINU_HUMAN | 655 | NSRKSLSPG | S |
| sp\|Q9UKV3\|ACINU_HUMAN | 657 | RKSLSPGVS | S |
| sp\|Q9UKV8\|AGO2_HUMAN | 387 | MRSASFNTD | S |
| sp\|Q9UKX7\|NUP50_HUMAN | 204 | QHGNSGRNS | S |
| sp\|Q9ULG6\|CCPG1_HUMAN | 188 | TVSASESED | S |
| sp\|Q9ULG6\|CCPG1_HUMAN | 186 | KKTVSASES | S |
| sp\|Q9ULH1\|ASAP1_HUMAN | 1008 | TGDVSPKAQ | S |
| sp\|Q9ULT8\|HECD1_HUMAN | 1386 | KGSSSSVCS | S |
| sp\|Q9ULV3\|CIZ1_HUMAN | 575 | RPSDSVSST | S |
| sp\|Q9ULV3\|CIZ1_HUMAN | 567 | TVPLTPVPR | T |
| sp\|Q9UMZ2\|SYNRG_HUMAN | 752 | FRQLSLEGS | S |
| sp\|Q9UQ35\|SRRM2_HUMAN | 1326 | LRENSFGSP | S |
| sp\|Q9UQ35\|SRRM2_HUMAN | 1320 | ELSNSPLRE | S |
| sp\|Q9UQ35\|SRRM2_HUMAN | 1522 | GSESSVDQK | S |
| sp\|Q9UQ35\|SRRM2_HUMAN | 857 | GSITSPQAN | S |
| sp\|Q9UQ35\|SRRM2_HUMAN | 1403 | NQSISSPVL | S |
| sp\|Q9UQ35\|SRRM2_HUMAN | 1404 | QSISSPVLD | S |
| sp\|Q9UQ35\|SRRM2_HUMAN | 1521 | SGSESSVDQ | S |
| sp\|Q9UQ35\|SRRM2_HUMAN | 1318 | HKELSNSPL | S |
| sp\|Q9UQ35\|SRRM2_HUMAN | 1453 | DGSGTPSRH | T |
| sp\|Q9UQ35\|SRRM2_HUMAN | 1413 | AVPRTPSRE | T |
| sp\|Q9UQ35\|SRRM2_HUMAN | 455 | REISSSPTS | S |
| sp\|Q9UQ35\|SRRM2_HUMAN | 1444 | RSGSSPGLR | S |
| sp\|Q9UQ35\|SRRM2_HUMAN | 456 | EISSSPTSK | S |
| sp\|Q9UQ35\|SRRM2_HUMAN | 780 | RRSLSGSSP | S |
| sp\|Q9UQ35\|SRRM2_HUMAN | 778 | SLRRSLSGS | S |
| sp\|Q9UQ35\|SRRM2_HUMAN | 774 | KSRLSLRRS | S |
| sp\|Q9UQ35\|SRRM2_HUMAN | 783 | LSGSSPCPK | S |
| sp\|Q9UQ35\|SRRM2_HUMAN | 1511 | NKCLTPQRE | T |
| sp\|Q9Y2H0\|DLGP4_HUMAN | 973 | VRQNSATES | S |
| sp\|Q9Y2I7\|FYV1_HUMAN | 1714 | PKSSSPIRL | S |
| sp\|Q9Y2K7\|KDM2A_HUMAN | 28 | DDGISDDEI | S |
| sp\|Q9Y2L6\|FRM4B_HUMAN | 608 | SVPHSPRIL | S |
| sp\|Q9Y2L6\|FRM4B_HUMAN | 662 | QGGRSMPTT | S |
| sp\|Q9Y2L6\|FRM4B_HUMAN | 778 | SNSGSMPNL | S |
| sp\|Q9Y2U8\|MAN1_HUMAN | 402 | FSVDSPRIY | S |
| sp\|Q9Y2V2\|CHSP1_HUMAN | 30 | SRERSPSPL | S |
| sp\|Q9Y2V2\|CHSP1_HUMAN | 32 | ERSPSPLRG | S |
| sp\|Q9Y2W1\|TR150_HUMAN | 377 | KEKGSFSDT | S |
| sp\|Q9Y2W1\|TR150_HUMAN | 379 | KGSFSDTGL | S |
| sp\|Q9Y2W1\|TR150_HUMAN | 248 | PRERSPALK | S |
| sp\|Q9Y2X3\|NOP58_HUMAN | 502 | EEPLSEEEP | S |
| sp\|Q9Y2X7\|GIT1_HUMAN | 388 | DSVASDEDT | S |
| sp\|Q9Y371\|SHLB1_HUMAN | 190 | ETRNSSEQE | S |
| sp\|Q9Y388\|RBMX2_HUMAN | 187 | QPSSSSPRR | S |
| sp\|Q9Y3E2\|BOLA1_HUMAN | 81 | FEGLSPLQR | S |
| sp\|Q9Y3P9\|RBGP1_HUMAN | 35 | QGDETPSTN | T |
| sp\|Q9Y3Q8\|T22D4_HUMAN | 165 | PQARSFTGG | S |
| sp\|Q9Y3Q8\|T22D4_HUMAN | 123 | AGGRSLDSR | S |
| sp\|Q9Y3Q8\|T22D4_HUMAN | 229 | SGARTPPLS | T |
| sp\|Q9Y3Q8\|T22D4_HUMAN | 211 | SRAATPLPS | T |
| sp\|Q9Y3T9\|NOC2L_HUMAN | 673 | DLNSSEEDD | S |
| sp\|Q9Y3T9\|NOC2L_HUMAN | 672 | FDLNSSEED | S |
| sp\|Q9Y4F5\|C170B_HUMAN | 655 | PVPGSPGGQ | S |
| sp\|Q9Y4H2\|IRS2_HUMAN | 1148 | RRRHSSETF | S |
| sp\|Q9Y4H2\|IRS2_HUMAN | 1156 | FSSTTTVTP | T |
| sp\|Q9Y4X5\|ARI1_HUMAN | 517 | ISQDSLQDI | S |
| sp\|Q9Y5K6\|CD2AP_HUMAN | 458 | LRPKSVDFD | S |
| sp\|Q9Y613\|FHOD1_HUMAN | 523 | LIPASPKAE | S |
| sp\|Q9Y6W5\|WASF2_HUMAN | 474 | ATILSRRIA | S |

1. Rusin SF, Schlosser KA, Adamo ME, Kettenbach AN: **Quantitative phosphoproteomics reveals new roles for the protein phosphatase PP6 in mitotic cells**. *Science Signaling* 2015, **8**(398):rs12.

2. Sarhan A, Szyroka J, Begum S, Tomlinson M, Hotchin N, Heath J, Cunningham D: **Quantitative Phosphoproteomics Reveals a Role for Collapsin Response Mediator Protein 2 in PDGF-Induced Cell Migration**. *Scientific Reports* 2017, **7**:3970.

3. Rusin SF, Adamo ME, Kettenbach AN: **Identification of Candidate Casein Kinase 2 Substrates in Mitosis by Quantitative Phosphoproteomics**. *Frontiers in Cell and Developmental Biology* 2017, **5**(97).

4. Kao L, Wang Y-T, Chen Y-C, Tseng S-F, Jhang J-C, Chen Y-J, Teng S-C: **Global analysis of cdc14 dephosphorylation sites reveals essential regulatory role in mitosis and cytokinesis**. *Mol Cell Proteomics* 2014, **13**(2):594-605.

5. Luo F, Wang M, Liu Y, Zhao X-M, Li A: **DeepPhos: prediction of protein phosphorylation sites with deep learning**. *Bioinformatics* 2019, **35**(16):2766-2773.
